# The crystal structure of dypingite: understanding the long-range disorder

**DOI:** 10.1107/S1600576725007915

**Published:** 2025-10-10

**Authors:** Anton Sednev-Lugovets, Yang Lu, Ørnulv Vistad, Patricia Almeida Carvalho, Alexander Missyul, Håkon Austrheim, Henrik Friis, Matylda N. Guzik

**Affiliations:** a Department of Technology Systems University of Oslo Gunnar Randers vei 19 Kjeller2007 Norway; b Natural History Museum University of Oslo Blindern Oslo1172 Norway; c SINTEF Industry, Forskningsveien 1, Oslo0374, Norway; d CELLS-ALBA Synchrotron, Carrer de la Llum 2-26, Barcelona08290, Spain; e Department of Geosciences University of Oslo Sem Saelands vei 1 Oslo0371 Norway; Australian Synchrotron, ANSTO, Australia

**Keywords:** dypingite, structural disorder, uniaxial elongation, magnesium hy­droxy carbonates, long-range disorder

## Abstract

Dypingite, a hydrated magnesium carbonate hydroxide mineral [Mg_5_(CO_3_)_4_(OH)_2_·*X*H_2_O, *X* = 5–6], exhibits promising catalytic and purification properties. Although it was discovered 55 years ago, the crystal structure of this compound has remained unknown due to its aggregated morphology and structural disorder. This work investigates the origin of this phenomenon through a systematic analysis of synthetic and natural mineral samples, using synchrotron powder X-ray diffraction, thermogravimetric analysis and transmission electron microscopy. The findings reveal that ambient humidity significantly influences dypingite’s structural properties at room temperature. High humidity (80% relative humidity at 22 °C) causes inhomogeneous expansion of the unit cell along the crystallographic *c* axis, leading to long-range structural disorder. Conversely, at 20% relative humidity at 22 °C, the mineral structure exhibits a shorter *c* lattice constant and reduced structural disorder. Chemical analysis reveals that samples kept at 80% and 20% relative humidity for 10 days differ by one molecule of water of hydration, yielding Mg_5_(CO_3_)_4.5(2)_(OH)_0.96(3)_·6.0(2)H_2_O and Mg_5_(CO_3_)_4.5(2)_(OH)_1.02(4)_·5.0(2)H_2_O, respectively. The results obtained demonstrate that the crystal structure of dehydrated dypingite [Mg_5_(CO_3_)_4.5(2)_(OH)_1.02(4)_·5.0(2)H_2_O] derives from hydro­magnesite’s unit cell tripled along the *a* axis. The analysis of the mineral crystal structure provides insight into the role of humidity on the structural properties of dypingite, including unit-cell dimensions and long-range disorder.

## Abstract 2

After 55 years, the average crystal structure of dypingite is finally revealed. Our research shows that ambient humidity controls long-range disorder within this mineral and unlocks its potential as a tunable, non-toxic material for developing next-generation functional materials.

## 1. Introduction

For a long time, the crystallography world perceived defects in crystal structures as imperfections and undesired traits. Nowadays, it is widely recognized that defects play a crucial role in determining physical and chemical properties of solid materials (Spitaler & Estreicher, 2018 ▸). The ability to engineer and control the level of disorder in material crystal structures offers the potential to advance technology fields through innovative solutions. The accurate management of defects and impurities in Ge and Si crystals at the beginning of the 20th century paved the way for modern electronics and computing (Sgourou *et al.*, 2022 ▸). Similarly, the in-depth understanding of the structural properties of the ceramic cuprate materials and the precise control of defects within their crystal structures led to advancements in the field of superconductors, providing high-precision MRI machines and large high-energy accelerators (Parizh *et al.*, 2017 ▸; Yao & Ma, 2021 ▸). In light of these advancements, numerous research groups have focused on studying disordered solid materials and exploring methods to engineer disorder within their crystal structures to gain control over the physical and chemical properties of the compounds (Chen, 2024 ▸; Yu *et al.*, 2021 ▸; Simonov & Goodwin, 2020 ▸). However, this can be achieved only through the comprehensive understanding of the material crystal structure. Unfortunately, the presence of disorder often results in structural complexity, making material characterization more difficult.

**Figure 1:**
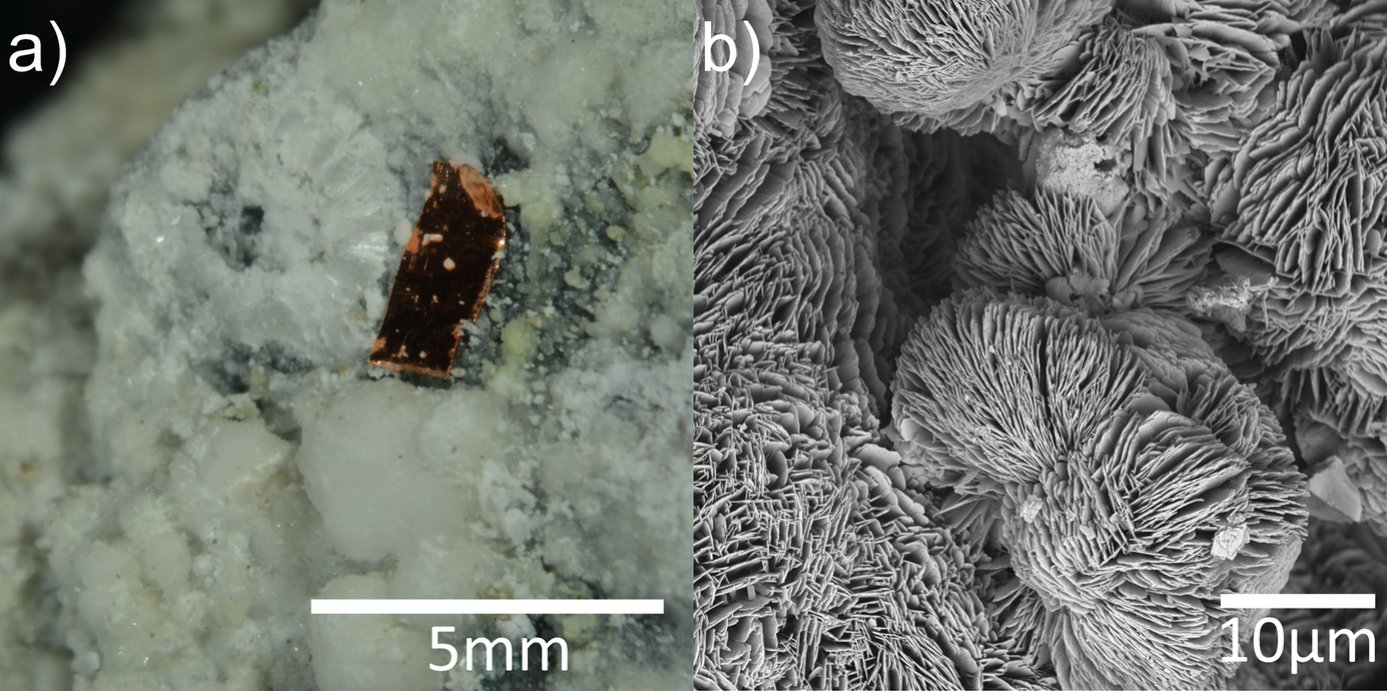
Naturally formed dypingite: (*a*) microphotograph of a dypingite layer on a serpentine rock; (*b*) SEM image of dypingite’s layers.

This is also the case for dypingite [Mg_5_(CO_3_)_4_(OH)_2_·*X*H_2_O, *X* = 5], which was discovered and identified in the 1970s by Gunnar Raade in the serpentine–magnesite deposit at Dypingdal, Snarum, South Norway (Raade, 1970 ▸). Dypingite plays a significant role in natural carbon sequestration processes. This mineral commonly forms on the surface of ultramafic rocks, particularly serpentine, through weathering reactions involving atmospheric CO_2_ and water (Boschi *et al.*, 2017 ▸; Power *et al.*, 2013 ▸; Raade, 2012 ▸; Harrison *et al.*, 2019 ▸). This natural carbonation process represents a long-term mechanism for CO_2_ mineralization, where carbon dioxide is permanently fixed in stable carbonate minerals (Power *et al.*, 2013 ▸; Kelemen *et al.*, 2019 ▸). Beyond that, dypingite has attracted attention due to its ability to form flower-like nanoparticles (nanoflowers; Fig. 1 ▸) with large external surface areas. This unique property makes dypingite a promising functional material for applications in catalysis and water filtration (Chinna Rajesh *et al.*, 2016 ▸; Rajesh *et al.*, 2013 ▸; Naqvi, 2014 ▸; Hamilton *et al.*, 2016 ▸), leveraging its high surface area and structural characteristics. Attempts to reproduce the mineral under laboratory conditions have led to the discovery of the one-pot synthesis of stable, non-toxic, environmentally friendly nanoparticles (Zhang *et al.*, 2006 ▸; Cheng *et al.*, 2009 ▸; Yang *et al.*, 2012 ▸; Hao *et al.*, 2009 ▸).

**Figure 2:**
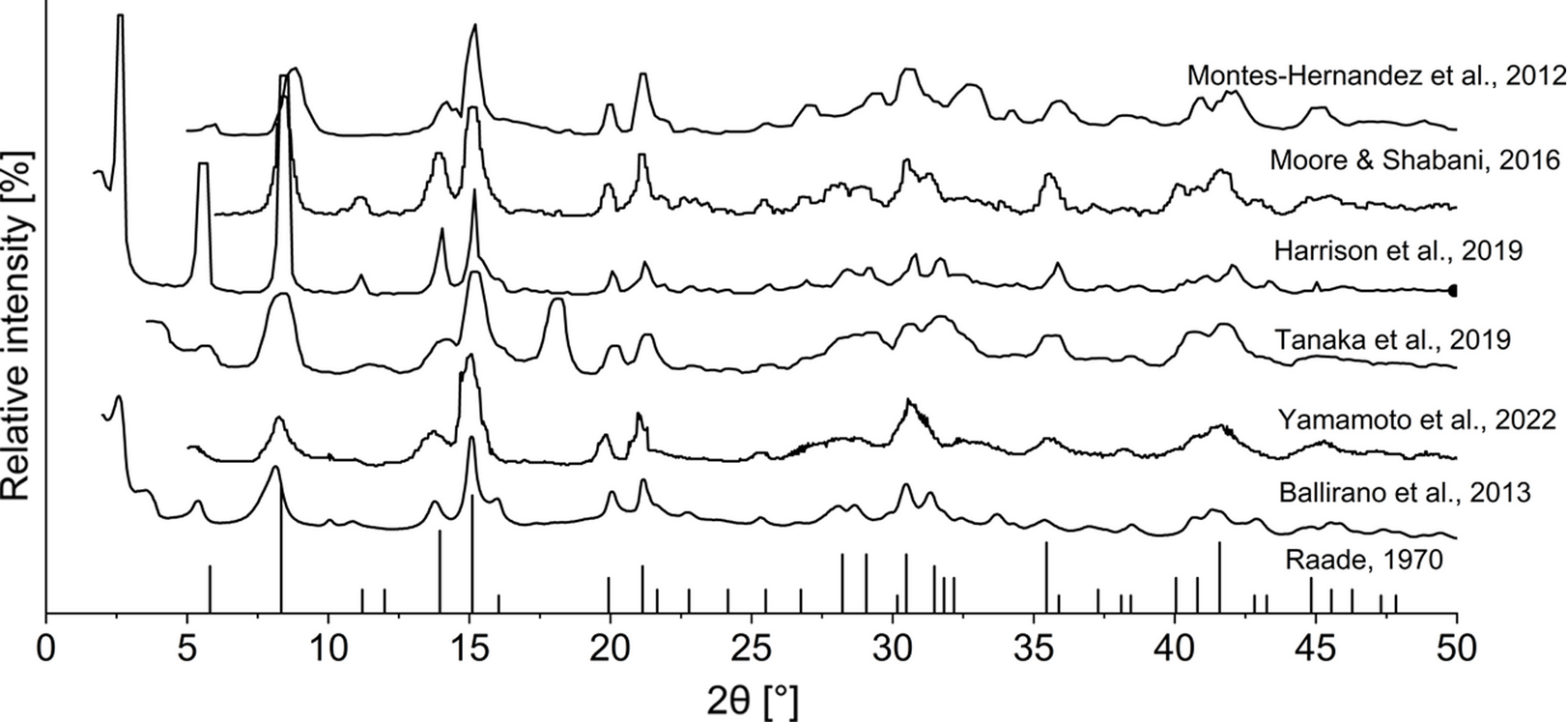
PXD patterns of synthetic dypingite reported in the literature. The diffraction patterns were digitized from the original papers and recalculated to the Cu *K*α_1_ radiation. Adopted with permission from Raade (1970 ▸), Ballirano *et al.* (2013 ▸), Yamamoto *et al.* (2022 ▸), Tanaka *et al.* (2019 ▸), Harrison *et al.* (2019 ▸), Moore & Shabani (2016 ▸) and Montes-Hernandez *et al.* (2012 ▸).

Even though dypingite nanoflowers have been reported as good catalysts (Chinna Rajesh *et al.*, 2016 ▸; Rajesh *et al.*, 2013 ▸) and water filters (Naqvi, 2014 ▸; Hamilton *et al.*, 2016 ▸), its crystal structure has remained unsolved due to the presence of aggregated morphology and complex structural disorder (Raade, 2012 ▸; Ballirano *et al.*, 2013 ▸). This is illustrated by the fact that variations in synthesis conditions led to a high level of diffuse scattering and inconsistencies in the position and shape of Bragg peaks, most notably the reflection at 8° 2θ in the reported powder X-ray diffraction (PXD) patterns (Fig. 2 ▸, Table 1 ▸) (Raade, 1970 ▸; Ballirano *et al.*, 2013 ▸; Yamamoto *et al.*, 2022 ▸; Tanaka *et al.*, 2019 ▸; Harrison *et al.*, 2019 ▸; Moore & Shabani, 2016 ▸; Montes-Hernandez *et al.*, 2012 ▸).

Interestingly, a closely related mineral, hydro­magnesite [Mg_5_(CO_3_)_4_(OH)_2_·*X*H_2_O, *X* = 4], whose nominal chemical composition differs from dypingite solely by the amount of crystallographic water, easily forms highly crystalline macromaterial. Thus, its chemical, physical and structural properties were reported a long time ago (Akao *et al.*, 1974 ▸). Moreover, the local atomic arrangements observed in the two compounds appear identical (Yamamoto *et al.*, 2022 ▸). Despite these similarities, all attempts to solve the crystal structure of dypingite have been unsuccessful. To the best of our knowledge, only one study has made progress in understanding of dypingite structural properties. Ballirano *et al.* (2013 ▸) successfully indexed the dypingite PXD pattern and concluded that the material crystallized with monoclinic symmetry [space group (SG): *P*2/*m*]. The authors also calculated the unit cell, which was a supercell of hydro­magnesite with the following lattice constants: 1*a* × 4*b* × 4*c*. However, the enormous volume of the as-calculated unit cell hindered further work on the material structure determination.

To understand factors that govern structural properties of synthetic and mineral dypingite, we formulated the following research questions to be answered during this study:

(1) What types of structural disorder are present in the dypingite crystal structure?

(2) What specific factors contribute to the observed structural disorder in dypingite?

(3) Can the degree of structural disorder in dypingite be controlled through experimental methods?

In this study, we have not only answered these questions and determined the average crystal structure of dehydrated dypingite but also uncovered a previously unknown ability of dypingite to change its unit-cell dimensions anisotropically in response to varying humidity. This property enables control over the structural disorder within the dypingite crystal, opening new avenues for potential applications of this intriguing mineral.

## 2. Experimental methods

For sample preparation, mineral dypingite was collected from the mine drift Konradine in the Feragen Ultramafic Body, 25 km east of Røros, Trøndelag, Norway. Synthetic dypingite was fabricated from commercially available MgCl_2_·6H_2_O (Sigma–Aldrich, ≥99%) and Na_2_CO_3_·10H_2_O (Sigma–Aldrich, ≥99.5%). The reagents were dissolved in deionized water, and the solutions obtained were mixed at room temperature (∼22 °C). The concentrations of Mg^2+^ and CO_3_^2−^ in the resulting mixture were 0.1 *M*. After 30 s of stirring, the mixture was heated to 40 °C and maintained at this temperature for 28 days, at atmospheric pressure. Subsequently, the material was filtered and kept at 80% relative humidity (RH) for 10 days. To investigate the nature of structural disorder in dypingite, samples with different hydration states were prepared. In this study, hydrated dypingite refers to the mineral or synthetic samples kept in a humidity-controlled cabinet at 80% RH and 22 °C for 10 days, until the Bragg peaks in the PXD patterns collected stopped changing positions. Dehydrated dypingite refers to synthetic dypingite stored in a desiccator (LLG GmbH, silica gel beads of 1–3 mm as a desiccant) at 20% RH and 22 °C for 10 days, ensuring no further changes in the diffraction peak positions were observed in the collected PXD data. The water content in both the hydrated and the dehydrated samples was determined from the results of thermogravimetric analysis (TGA) and differential scanning calorimetry (DSC) measurements.

TGA coupled with DSC (TGA–DSC) was performed using a NETZSCH STA 449F1 thermal analyzer. A sample of synthetic dypingite with a mass of 20 mg was inserted into an Al_2_O_3_ crucible, heated from 20 to 900 °C and then cooled to room temperature, both at a rate of 5 °C min^−1^ with a dwell time of 1 h at 900 °C. Before each measurement, the instrument was calibrated to account for the buoyancy effect. To exclude the possible influence of water adsorbed by the crucible surface, the holder was annealed at 1000 °C for 1 h before the measurement. All the experiments were performed under nitro­gen gas atmosphere at a flow rate of 50 ml min^−1^. The TGA–DSC measurements were repeated three times for all samples studied. The obtained TGA curves were used to calculate the chemical compositions of the hydrated and dehydrated dypingite samples. For this purpose, the TGA plots were divided into two zones, the low-temperature zone, associated with the dominant release of H_2_O (*T* < 433 °C), and the high-temperature zone (*T* > 433 °C), where the CO_2_ release was dominant. The onset temperature was determined from the intersection of two tangential lines drawn at the points of the fastest weight loss (see the example in Fig. S1 of the supporting information for the dehydrated dypingite sample). Since the residual powder after the TGA–DSC experiments contained exclusively MgO (Fig. S2), the weight of this phase in the studied samples was assumed to be equal to the weight of the residue. The MgO:CO_2_:H_2_O ratios determined for the hydrated and dehydrated samples, together with the calculated chemical compositions and estimated standard deviations, are listed in Table S1 of the supporting information. The Mg content was assumed to be the same for all the samples and equal to 5.

Synchrotron radiation PXD (SR-PXD) measurements were performed on dypingite samples at two different synchrotron facilities. Mineral dypingite was studied at the BL04-MSPD beamline at the ALBA synchrotron (Spain) using a diffraction instrument equipped with a high-throughput MYTHEN position-sensitive detector (λ = 0.95305 Å, 1.1 < 2θ, ° < 89.2, step size 0.006° 2θ). Measurements were performed with samples sealed in 0.7 mm boron glass capillaries. The wavelength was calibrated using a silicon standard (SRM 640e).

The synthetic hydrated and dehydrated dypingite samples were investigated at BM01 of the Swiss–Norwegian Beamline, ESRF (France), with a diffraction instrument equipped with a Dectris Pilatus 2M area detector (λ = 0.71073 Å, 0.005 < 2θ, ° < 35.68, step size 0.011° 2θ) (Dyadkin *et al.*, 2016 ▸). Measurements were performed on powders sealed in boron glass capillaries with an internal diameter of 0.5 mm. The wavelength was calibrated using LaB­_6_ (NIST 660b standard). Integration of the 2D diffraction images was done using the *Bubble* software (Dyadkin *et al.*, 2016 ▸; Black *et al.*, 2011 ▸). The samples for both synchrotron facilities were prepared without preliminary grinding, and any contact with solvents (water, ethanol *etc*.) was avoided. For easy comparison with the reported literature data, all collected SR-PXD patterns presented in this work were recalculated to Cu *K*α­_1_ radiation.

Transmission electron microscopy (TEM) experiments were performed on the hydrated dypingite samples with a Titan G2 60–300 microscope. The measurements were carried out at 60 kV with the TEM camera cooled by liquid nitro­gen to −196 °C. During the measurements, the crystallinity of the dypingite sample quickly deteriorated due to beam damage, resulting in the appearance of amorphous rings and the disappearance of diffraction spots. The morphology and particle sizes of the synthetic and mineral dypingite were measured by scanning electron microscopy (SEM) with an FEI Nova NanoSEM 650 instrument, with the electron beam voltage 350 V to 5 kV. The electron diffraction images of dehydrated dypingite were calculated using the *JEMS* software (JEMS-SWISS, Jongny, Switzerland).

SR-PXD patterns were indexed with *TOPAS* (version 6 software; Coelho, 2003 ▸, 2018 ▸). Profile analysis was performed using the Le Bail method, with peak shapes modeled by the Cagliotti function. The instrumental peak profile parameters (*U*, *V*, *W*) and asymmetry parameters (*X*, *Y*) were determined through whole pattern fitting of a LaB_6_ standard reference material. The refined profile parameters were subsequently applied during both Le Bail and Rietveld refinements. The unit-cell parameters obtained from the Le Bail profile analysis were used as initial values in the Rietveld refinement of dehydrated dypingite.

A crystal structure refinement was performed using a 3*a* × 1*b* × 1*c* structural model of hydro­magnesite with the space group *P*2_1_ generated by the *ISODISTORT* software (Campbell *et al.*, 2006 ▸). The Rietveld refinements of the crystal structure model were performed using the open-source software package *GSAS-II* (Toby & Von Dreele, 2013 ▸). The results obtained indicated a highly disordered crystal structure, making refinement of the atomic positions impossible without geometrical restraints. Therefore, soft (penalty) restraints for bond lengths in CO_3_ planar triangles and MgO_6_ octahedra were imposed, with a weight factor of 2, and were gradually relaxed to a value of 1 in the final stages of the refinement process. To account for the *hkl*-dependent diffuse scattering, caused by the long-range structural disorder in the studied material, the generalized Stephens model of the anisotropic peak broadening for monoclinic symmetry was applied. The initial estimates of the nine anisotropic broadening parameters were determined according to the procedure recommended by Stephens (1999 ▸). The total number of observed data points amounted to 3264, and the total number of refined parameters was 270, 243 of which were atomic coordinates (crystallographic positions of hydrogen atoms were excluded from the refinement), 15 background coefficients, 4 unit-cell parameters, 7 coefficients of the Stephens model and 1 histogram scale factor. All atomic site occupancies were fixed to 100%. The isotropic atomic displacement parameters (*U*_iso_) were constrained to 0.01 Å^2^ for all atomic positions and were not refined during the analysis.

## 3. Results and discussion

### 3.1. Structural properties of mineral and synthetic dypingite based on the SR-PXD data analysis

**Figure 3:**
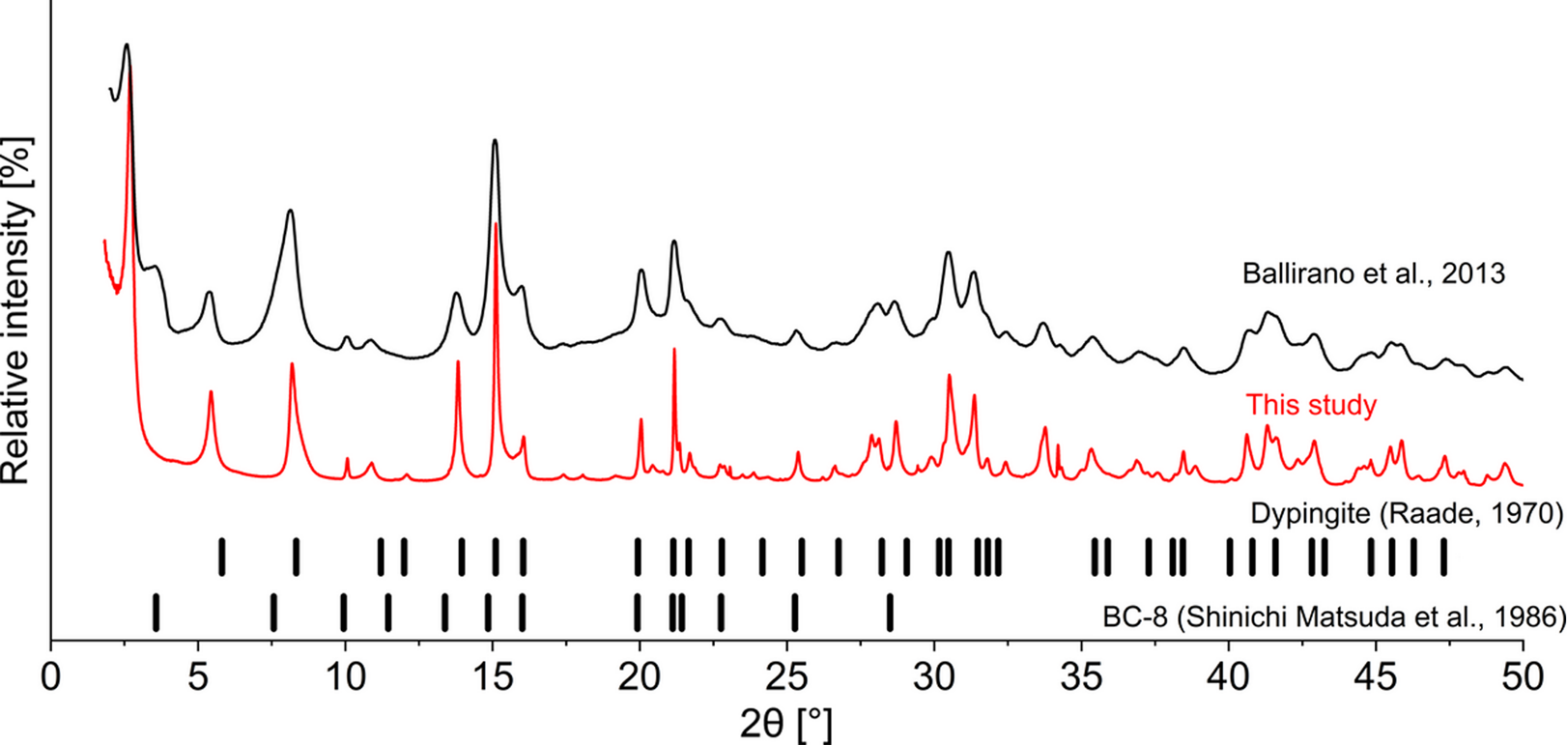
SR-PXD pattern of mineral dypingite collected in the present study (red) compared with Bragg peak positions of the dypingite-type mineral (vertical black lines), the BC-8 phase (Matsuda *et al.*, 1986 ▸) and PXD patterns collected by Ballirano *et al.* (2013 ▸) (black); λ = Cu *K*α_1_. Adopted with permission from Raade (1970 ▸), Ballirano *et al.* (2013 ▸) and Matsuda *et al.* (1986 ▸).

Fig. 3 ▸ presents Bragg peak positions of a dypingite-type material (Raade, 1970 ▸) and the PXD pattern of a dypingite sample synthesized for 230 days at 27 °C (Ballirano *et al.*, 2013 ▸), alongside the synchrotron powder X-ray diffraction data of the mineral sample collected in the present study. The latter demonstrates a significantly higher signal-to-noise ratio and better sample material crystallinity, marked by sharp and narrow Bragg peaks, which allows even weak reflections to be resolved, enabling reliable pattern indexing. Notably, our SR-PXD pattern lacks the 4° 2θ reflection observed in the Ballirano *et al.* (2013 ▸) data. This peak is attributed to a low-temperature metastable magnesium hy­droxy­carbonate phase [BC-8; (Matsuda *et al.*, 1986 ▸)] that can form below 30 °C.

**Figure 4:**
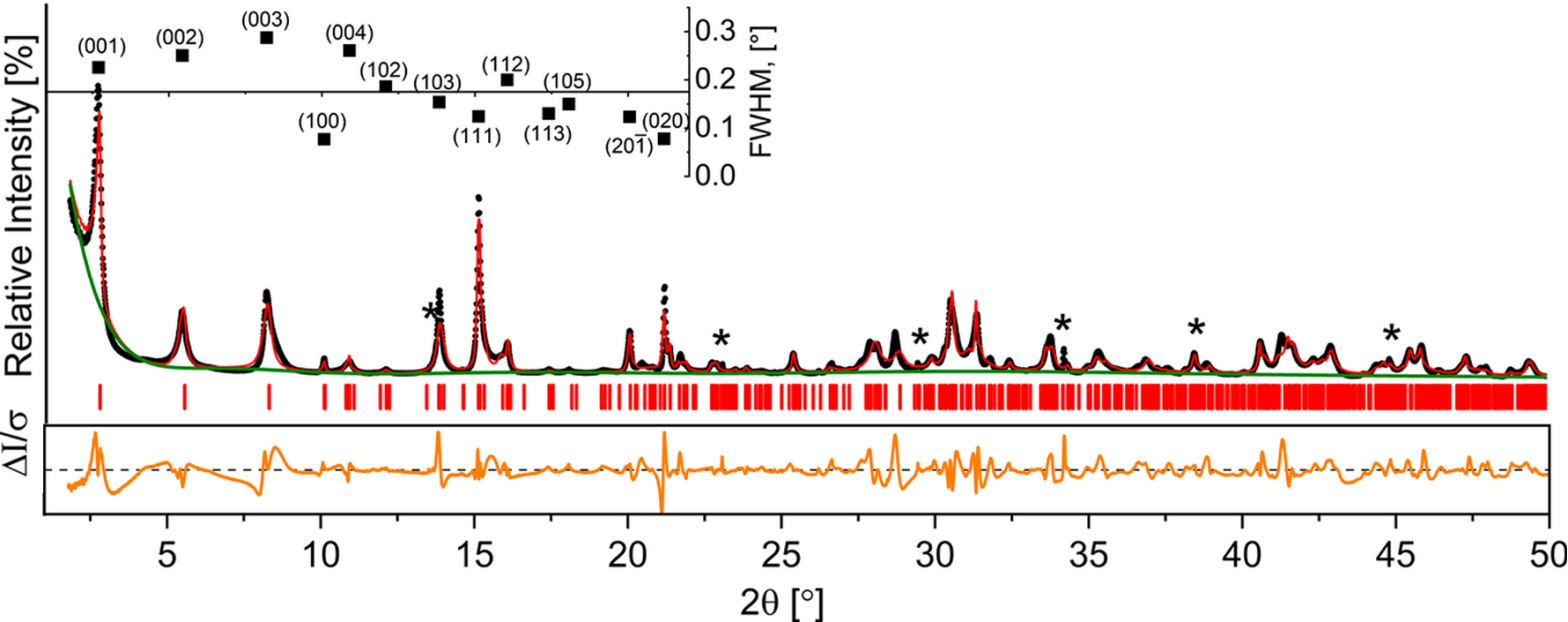
Results of the Le Bail profile refinement for mineral dypingite (SG *P*2_1_, λ = Cu *K*α_1_). Black, red and green lines represent experimental, calculated and background SR-PXD plots, respectively, while vertical red lines indicate the Bragg peak positions. The bottom line represents the difference between the experimental and calculated intensities. The inset shows the FWHM variations for the first 13 Bragg reflections. The Bragg peaks of nesquehonite (MgCO_3_·3H_2_O) are marked with an asterisk.

The profile analysis of the SR-PXD data suggests that the mineral has a monoclinic crystal structure and crystallizes in either the *P*2_1_ or the *P*2 SG. The refined unit-cell volume was approximately 2200 Å^3^ for both SGs. Notably, the results for the *P*2/*m* SG were exclusively associated with unit-cell volumes exceeding 10000 Å^3^. Such a significant volumetric discrepancy made the *P*2/*m* assignment incompatible with the experimental data obtained. Thus, subsequent unit-cell refinements were carried out exclusively on smaller configurations. Results of the Le Bail profile refinement for space group *P*2_1_ are depicted in Fig. 4 ▸, and the refinement for *P*2 can be found in Fig. S3.

The goodness-of-fit (χ^2^) values as well as the unit-cell parameters obtained from the profile refinements (Table 2 ▸) for the two tested SGs were almost identical, indicating that the correct lattice symmetry could not be determined exclusively by the analysis of the powder diffraction profiles (Toby, 2006 ▸). The calculated unit-cell parameters reveal a strong correlation with those of hydro­magnesite, as demonstrated by the following relationships:
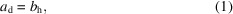

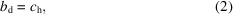

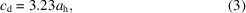
where *a*_d_, *b*_d_, *c*_d_ and *a*_h_, *b*_h_, *c*_h_ represent unit-cell parameters of dypingite and hydro­magnesite, respectively. Equation (3) implies that the dypingite unit cell may be constructed by a threefold enlargement of hydro­magnesite unit cell, rather than its 16-fold increase as suggested by Ballirano *et al.* (2013 ▸). The SG and crystallographic angles determined in this work for dypingite are different from those reported for hydro­magnesite, which indicates a more intricate relationship between the crystal structures of these two compounds.

A detailed analysis of the Bragg peak indices and their corresponding full width at half-maximum (FWHM) values reveals an interesting correlation. The observed 00*l* reflections (inset in Fig. 4 ▸) are broad and asymmetric, whereas 100, 020 and 201 are narrow and sharp. This observation suggests that the structural disorder present in the dypingite crystal spreads exclusively along the crystallographic *c* axis. This hypothesis is elaborated and further supported in the following sections.

While the SR-PXD patterns collected for mineral dypingite provided crucial information on its unit-cell parameters and valuable insight into the potential distribution of disorder in the material crystal structure, the presence of a secondary phase (nesquehonite) in the studied samples hindered the process of crystal structure determination. Given that all analyzed mineral samples were confirmed to contain impurities, we decided to synthesize a single-phase material for more detailed structural characterization.

**Figure 5:**
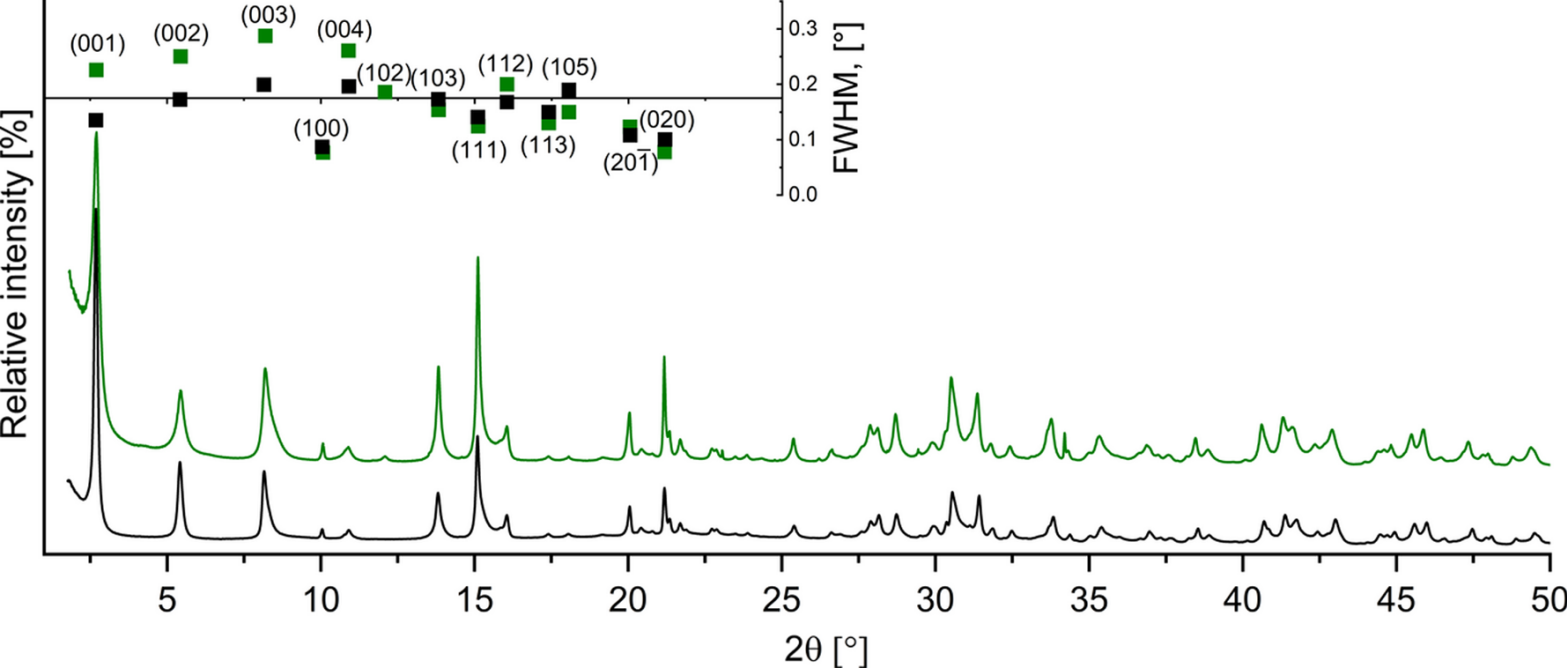
SR-PXD patterns of synthetic (blue) and mineral (green) dypingite with the inset showing the FWHM variations for the first 13 Bragg reflections; λ = Cu *K*α_1_.

The phase-pure dypingite sample was synthesized at 40 °C for 28 days at atmospheric pressure and was subsequently kept at 80% RH for 10 days. The microstructural analysis of the obtained material confirmed the formation of large spherical nanoflowers, ranging from 50 to 100 µm in diameter (Fig. S4). The comparative analysis of the SR-PXD patterns of the synthetic and mineral dypingite in Fig. 5 ▸ demonstrates close similarities between the intensities and positions of the Bragg peaks in the two datasets. However, the pattern of the synthetic dypingite shows lower-intensity background and narrower 00*l* diffraction reflections (inset in Fig. 5 ▸). This indicates better crystallinity of the synthetic dypingite sample than its mineral counterpart. For this reason, the as-synthesized powder was used for further structural analysis.

### 3.2. Influence of humidity on structural properties of synthetic dypingite

To further investigate the hypothesis that disorder in the dypingite crystal structure is present along the crystallographic *c* direction, synthetic dypingite was exposed to environments with varying humidity levels: 80% and 20% RH. For clarity, the samples maintained at 80% RH will be referred to as ‘hydrated dypingite’, while those exposed to 20% RH will be termed ‘dehydrated dypingite’.

**Figure 6:**
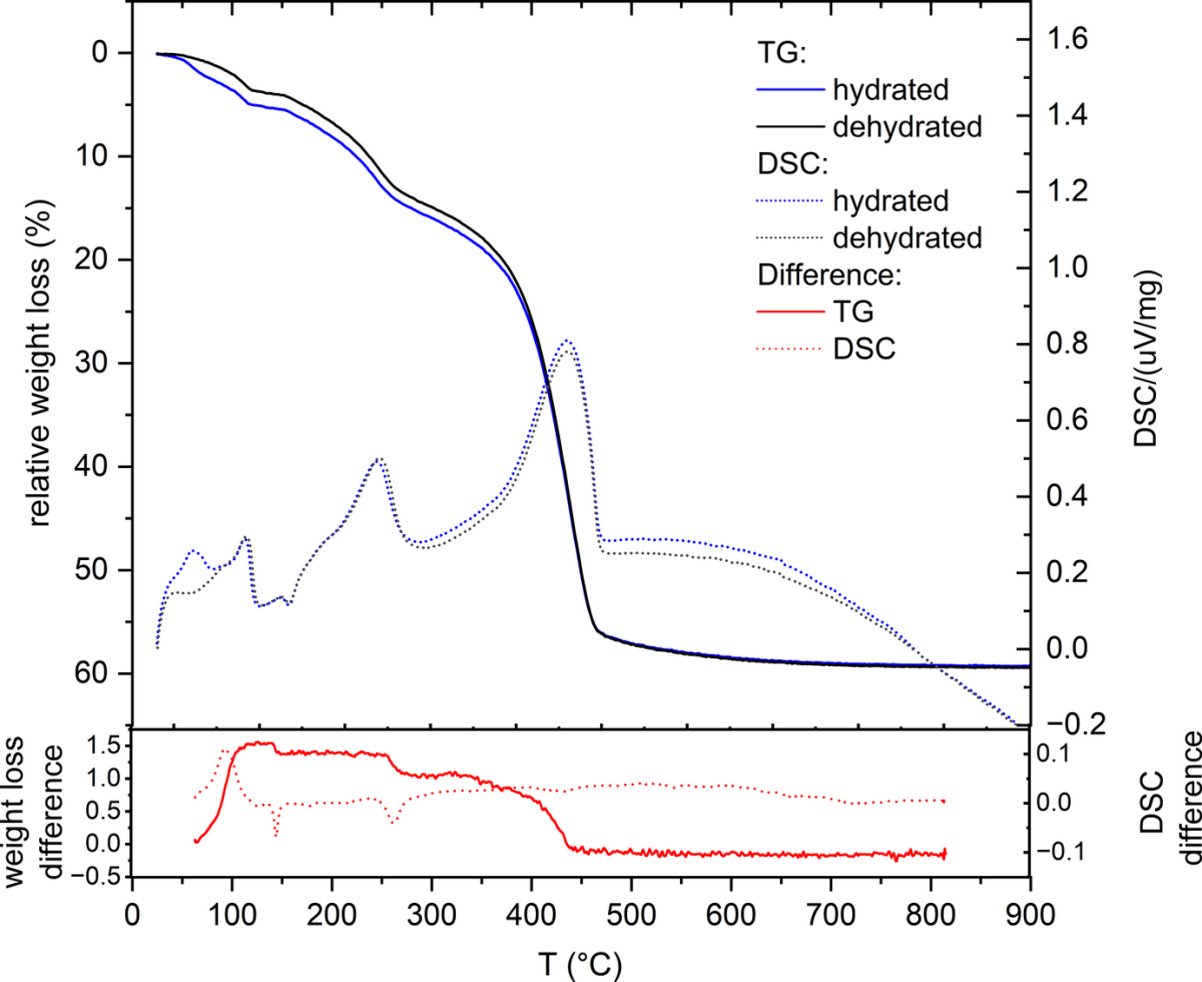
TGA–DSC results for the hydrated (blue lines) and dehydrated (black lines) synthetic dypingite. Solid lines represent TGA curves, whereas dashed lines correspond to DSC curves. The red lines at the bottom represent the subtraction of the results for dehydrated dypingite from the data collected for hydrated dypingite.

The compositional difference between the hydrated and dehydrated samples of synthetic dypingite was studied by thermogravimetric analysis, coupled with differential scanning calorimetry (TGA–DSC). On heating, the two samples underwent similar decomposition stages (Fig. 6 ▸) with peak temperature (*T*_p_) events at 120 and 245 °C, corresponding to the release of water from the material. The third process at *T*_p_ = 433 °C was associated with the sample decarbonation. While these findings are consistent with previous reports, the TGA–DSC curve of the hydrated sample demonstrated an additional decomposition step, with a pronounced exothermic effect at *T*_p_ = 62 °C (Raade, 1970 ▸; Suzuki & Ito, 1973 ▸; Canterford *et al.*, 1984 ▸; Yamamoto *et al.*, 2022 ▸; Frost *et al.*, 2008 ▸). This was associated with desorption of water previously accommodated by the sample during the hydration process.

**Figure 7:**
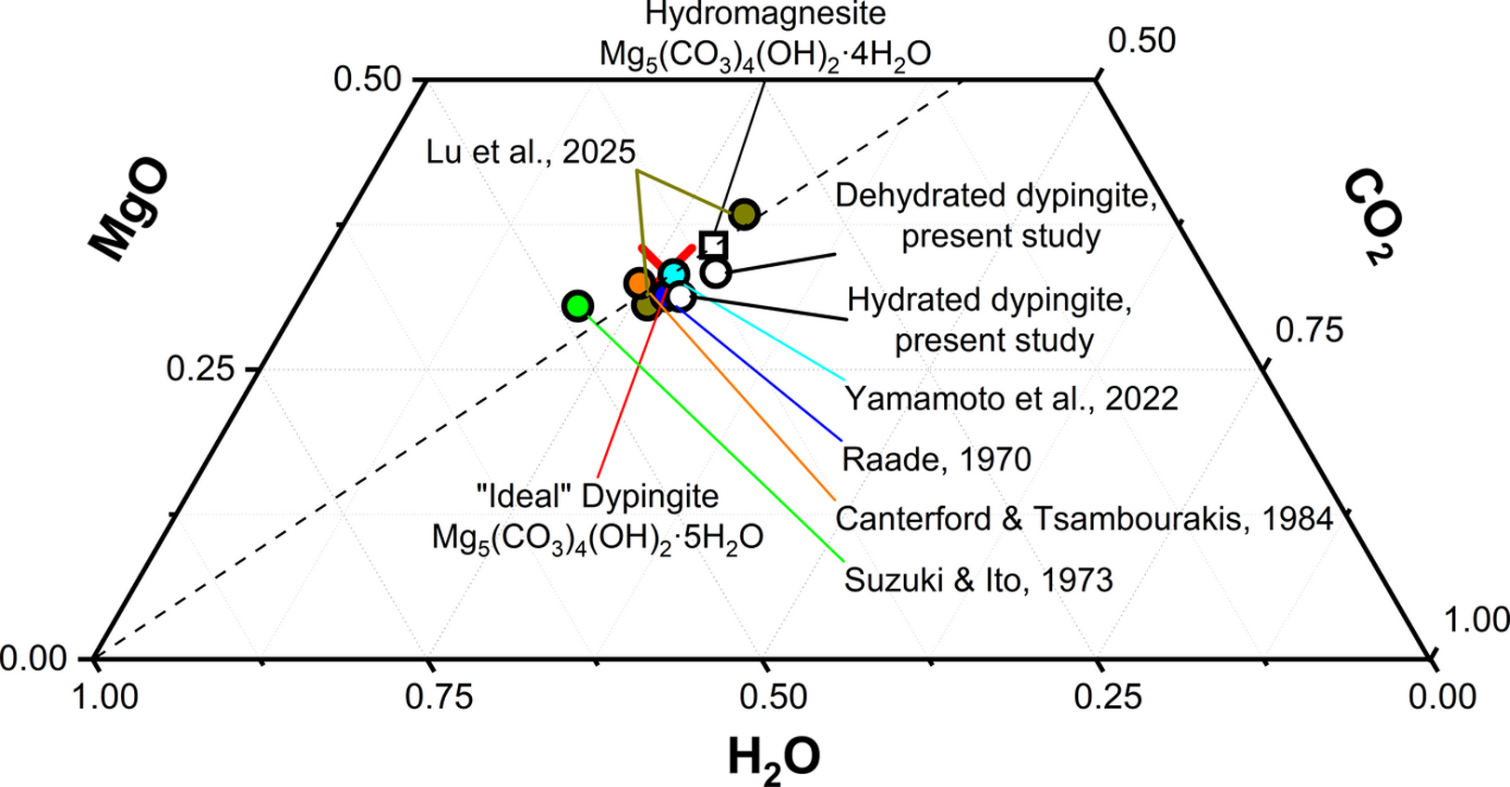
Ternary compositional diagram summarizing the dypingite chemical composition reported in the literature.

A detailed description of the TGA curve examination for both hydrated and dehydrated samples is provided in the experimental section and illustrated in Figs. S1 and S2 and Table S1. This analysis quantified the specific amount of H_2_O and CO_2_ released during the compound decomposition, and the mass of MgO left after the TGA–DSC experiment. For the dehydrated sample, the weight percentages of H_2_O, CO_2_ and MgO amounted to 19.9 (7), 39.6 (15) and 40.4 (15)%, respectively. For comparison, the hydrated sample exhibited the following weight percentages: 22.5 (8)% of CO_2_, 38.5 (14)% of H_2_O and 39.0 (14)% of MgO. The obtained results are in good agreement with values reported earlier in the literature, as demonstrated in Fig. 7 ▸ and summarized in Table S2.

On the basis of the TGA–DSC results obtained in the present study, the following chemical compositions of the hydrated and dehydrated dypingite have been proposed: Mg_5_(CO_3_)_4.5(2)_(OH)_1.02(4)_·5.0(2)H_2_O and Mg_5_(CO_3_)_4.5(2)_(OH)_0.96(3)_·6.0(2)H_2_O, respectively. These values indicate that the difference between water content in the two materials is 1.0 (2). Interestingly, the weight loss discrepancy between the two samples at 100 °C is 3.4%, as shown in Fig. 6 ▸ (red solid line at the bottom), corresponding to 0.97 molecules of water. Thus, one can conclude that the additional water accommodated by the compound during the hydration process (hereafter referred to as extra-water) is weakly bonded and completely released on heating to 100 °C. However, this observation raises the question of whether the extra-water was adsorbed only by the material surface or incorporated into its crystal structure.

**Figure 8:**
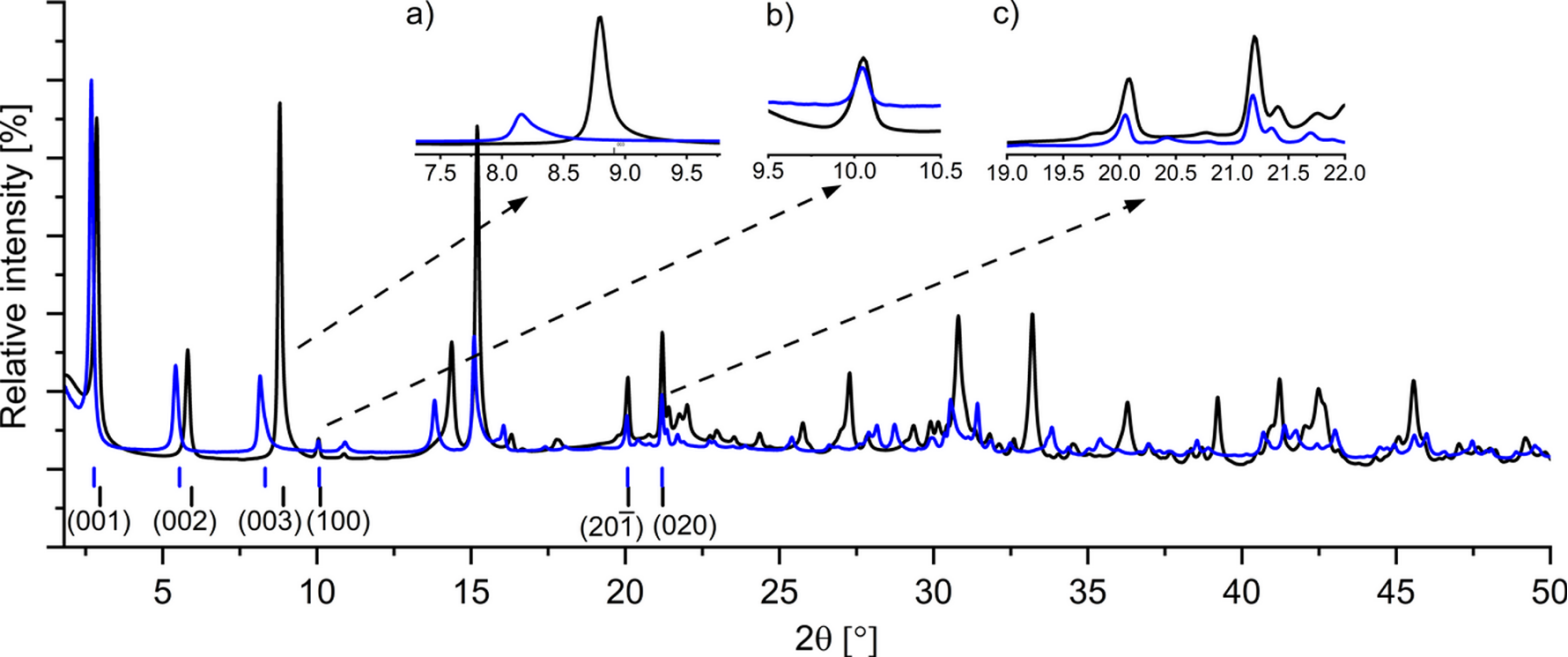
SR-PXD patterns of the hydrated (blue) and dehydrated (black) dypingite, λ = Cu *K*α_1_.

Two facts support the latter. Firstly, our previous research on mineral dypingite, combined with our current observations of synthetic dypingite (Figs. S5 and S6), has indicated that the hydration process of this compound is completely reversible (Lu *et al.*, 2023 ▸, 2025 ▸). This transition between fully hydrated and fully dehydrated states occurs within a span of 10 days. Secondly, variation in the humidity level surrounding the dypingite samples resulted in modifications of intensities, positions and broadness of selected Bragg peaks in the collected diffraction data (Fig. 8 ▸). As such, the observed alterations can be attributed to the incorporation of the additional water molecules into the compound crystal structure.

To identify specific changes in the dypingite unit cell due to hydration, we performed Le Bail profile refinements of the SR-PXD data for both the water-rich and the water-depleted samples. Analysis of the data (Table 3 ▸) demonstrated that the variations in the *c* lattice parameter (2.060 Å) during the process were larger than those observed for the *a* (0.086 Å) and *b* (0.0032 Å) constants. This anisotropic unit-cell expansion resulted in a shift of the 001, 002 and 003 Bragg reflections toward lower 2θ angles (Fig. 8 ▸). We will refer to the changes in the Bragg positions of these three diffraction peaks as changes in the 00*l* reflections because it is expected that the entire 00*l* family of Bragg reflections is affected by the changes in the *c* parameter.

Despite the changes in the 00*l* diffraction peaks, the 100, 20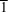 and 020 reflections remained unchanged (Fig. 8 ▸). Moreover, all noticeable 00*l* peaks became broader and more asymmetric after the sample hydration (*e.g.* FWHM of 003 = 0.200° versus 0.138° for the hydrated and dehydrated phase, respectively), while the broadness of the 100, 20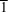 and 020 reflections remained unaffected by the humidity variations (*e.g.* FWHM of 020 = 0.1550° versus 0.1547° for the hydrated and dehydrated phase, respectively). Therefore, one can infer that the incorporation of extra-water into the dypingite crystal structure occurs along the *c* axis. This leads to uniaxial unit-cell enlargement and, in turn, the formation of the structural disorder in this direction.

The presence of disorder in the dypingite crystal structure arises from the uneven distribution of the water molecules incorporated during the material hydration. This results in elongation of the *c* lattice parameter to 32.101 (5) Å (Table 3 ▸). Conversely, the dehydration process removes the extra-water, causing the compound to shrink in the *c*-axis direction with a unit-cell volume decrease of 160.5 Å^3^ (Table 3 ▸). When calculated per formula unit, these difference translates into 1.12 (9) water molecules [using a molar volume of 24 (2) Å^3^ for water (Glasser, 2019 ▸)], which matches well the water content difference found by TGA. The observed *c* parameter value of 30.047 (9) Å is equivalent to the tripled parameter *a* of the hydro­magnesite unit cell. However, the angles and space groups of these two phases differ, indicating that their structural relationship is more complex than a simple supercell transformation. To address this issue, the group–subgroup relations between the lattices of dehydrated dypingite and hydro­magnesite have been explored.

By implementing the approach employed in the *ISODISTORT* software, the lower-symmetry lattices (*P*2 or *P*2_1_) were investigated, with the hydro­magnesite supercell (3*a* × 1*b* × 1*c*) used as a prototype crystal structure (Campbell *et al.*, 2006 ▸). While the search over arbitrary *k*-points did not return any solution for the *P*2 SG, the lattice constants generated for the *P*2_1_ symmetry were similar to those obtained through the indexing of SR-PXD data of the dehydrated synthetic dypingite (Table 3 ▸). By applying the following transformation matrix to the hydro­magnesite supercell (3*a ×* 1*b* × 1*c*),
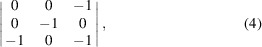
and by shifting the unit cell origin to (0, 0, 1/4) the *P*2_1_ subgroup of the hydro­magnesite supercell (3*a* × 1*b* × 1*c*) was obtained. The generated atomic positions, listed in Table S2, were used for further refinement of the crystal structure of dehydrated dypingite. The chemical composition of this crystal structure model is identical to that of hydro­magnesite [Mg_5_(CO_3_)_4_(OH)_2_·4H_2_O]. The structural model for dehydrated dypingite having been proposed, in the next step the refinement and validation processes of the suggested crystal structure can be carried out.

### 3.3. Crystal structure of the dehydrated dypingite versus varying humidity levels

**Figure 9:**
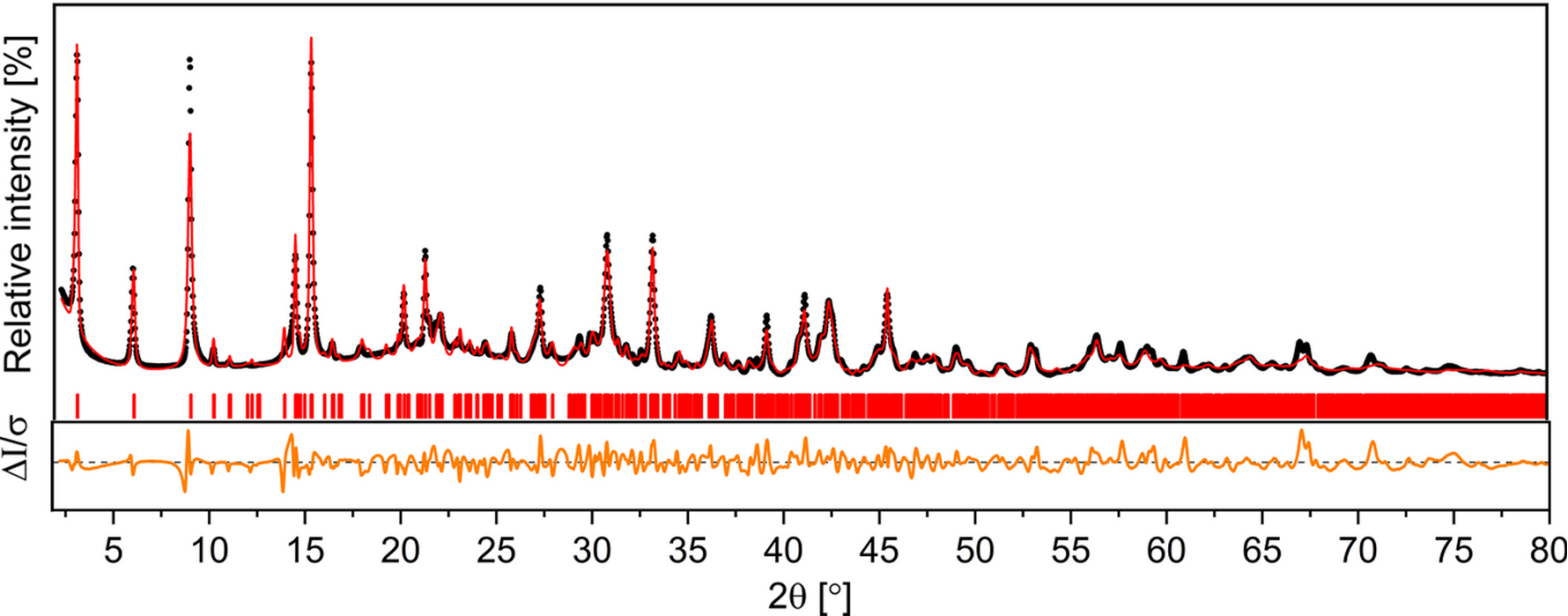
Graphical representation of the Rietveld refinement results obtained for the synthetic dehydrated dypingite. Black dots, red lines and vertical red bars represent the experimental and calculated powder diffraction profiles and the calculated positions of the Bragg peaks, correspondingly. The orange line illustrates the difference between experimental and calculated SR-PXD data; λ = Cu *K*α_1_.

The crystal structure model of dehydrated dypingite was refined using the Rietveld method. The initial and refined values of the atomic coordinates and unit-cell parameters are listed in Tables S3 and S4, respectively. The graphical results of the performed Rietveld analysis are presented in Fig. 9 ▸. The refinements resulted in a weighed profile *R* factor (*R*_wp_) of 9.80%, with a profile expected value (*R*_exp_) of 1.40% and goodness-of-fit (χ^2^) of 7.00, indicating a good agreement between the experimental and calculated SR-PXD patterns.

**Figure 10:**
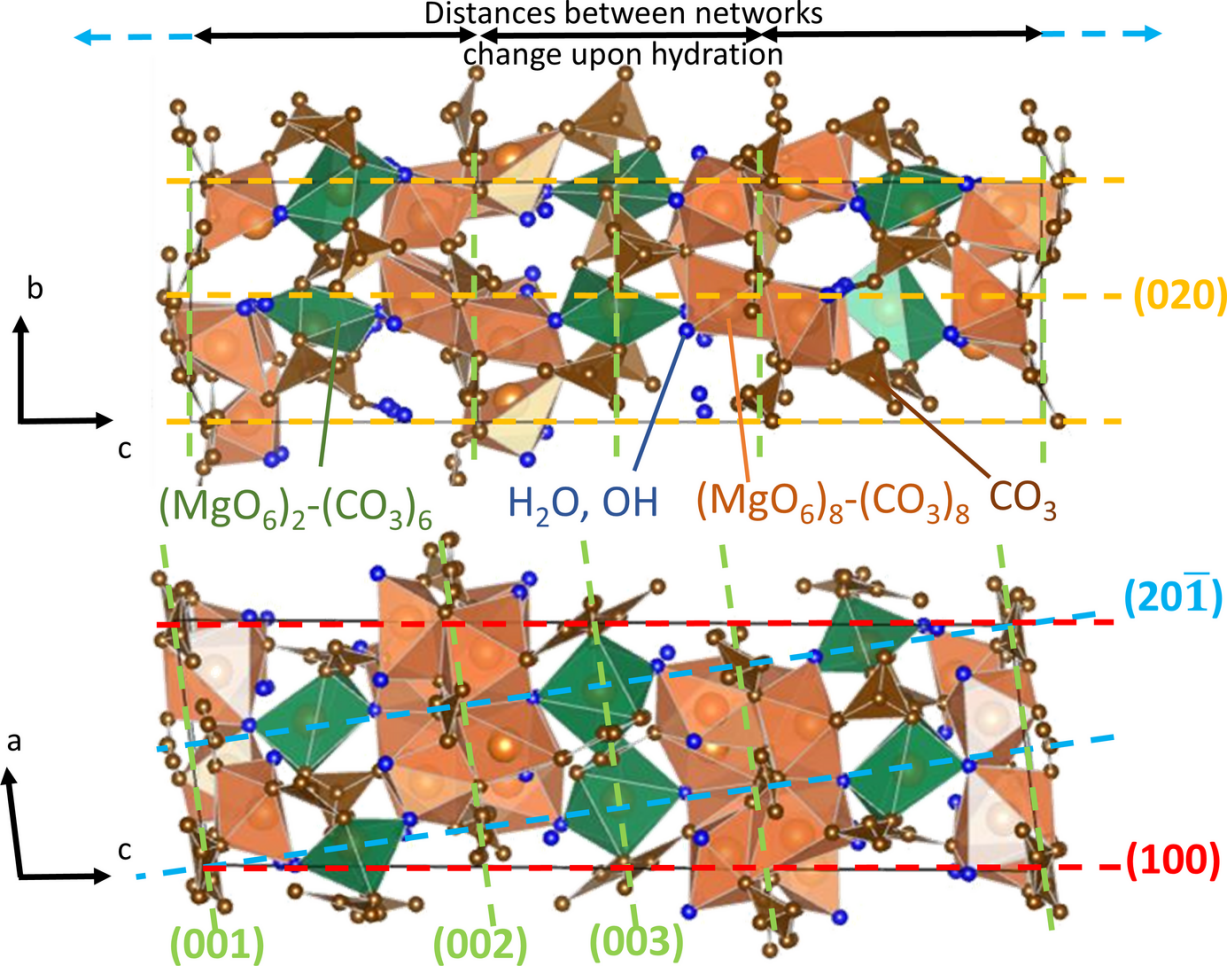
Refined crystal structure of dehydrated dypingite presented perpendicular to the *bc* (top) and *ac* (bottom) axes.

**Figure 11:**
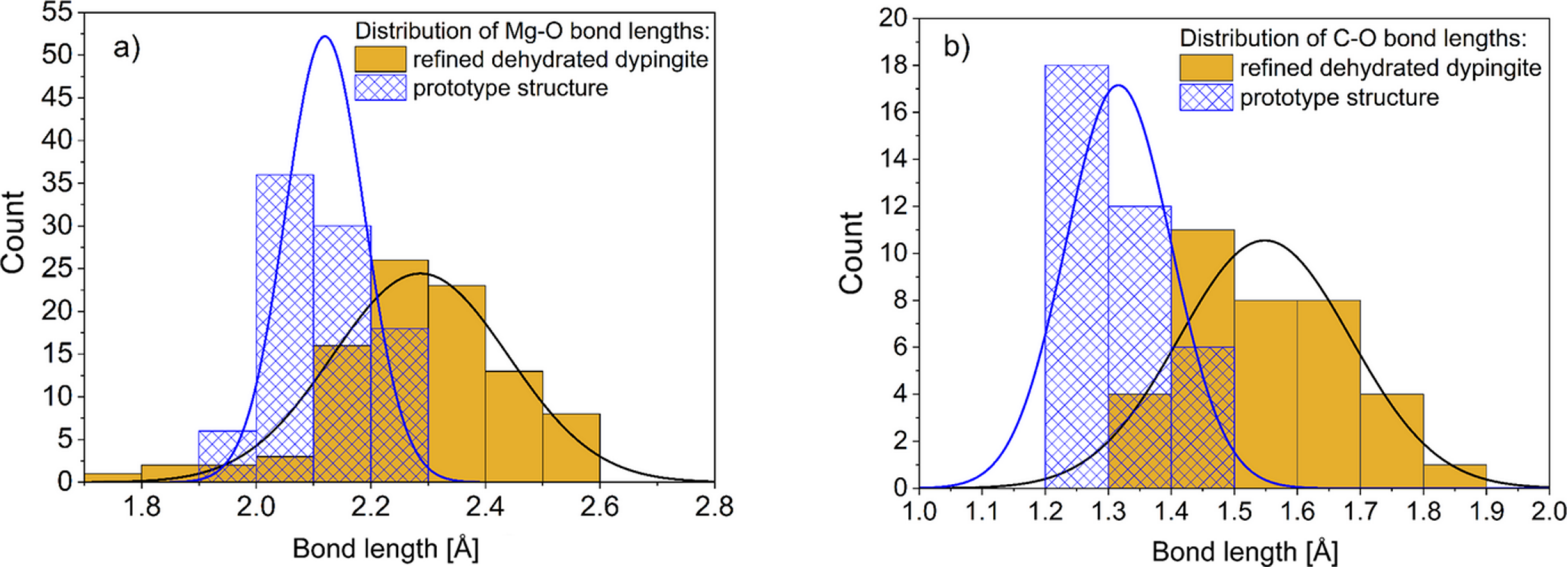
Distribution of the (*a*) Mg–O and (*b*) C–O bond lengths in the prototype and refined crystal structure of dehydrated dypingite.

The final refined crystal structure can be described as *distorted* hydro­magnesite. Fig. 10 ▸ illustrates alternating layers of (MgO_6_)_8_–(CO_3_)_8_ and (MgO_6_)_2_–(CO_3_)_6_ networks that resemble those found in the hydro­magnesite crystal structure. The refinement statistics (χ^2^ = 7.00) reflect the inherent complexity of this system. The crystal structure of dehydrated dypingite exhibits a certain extent of disorder leading to diffuse electron density distribution, which prevents the refinement process from converging. Thus, to achieve a robust structural model, certain modeling compromises were necessary, including the omission of hydrogen atoms and the application of geometrical restraints for bond lengths in the CO_3_ planar triangles and the MgO_6_ octahedra. While these adjustments contributed to the elevated refinement statistics, they preserved the integrity of the core structural model. A comparison of Mg—O and C—O bond lengths between the refined structure and the prototype structure (Fig. 11 ▸) reveals subtle geometric distortions in the MgO_6_ octahedra and CO_3_ planar triangles from their ideal configurations. These characteristics are common for magnesium carbonates and reflect a structural complexity of the system (Bischoff *et al.*, 1985 ▸; Deelman, 2021 ▸). The rotational and positional disorder of MgO_6_ octahedra and CO_3_ planar triangles result in an average crystal structure that may not accurately represent the true local atomic arrangements. Note that the refined structural model assumes the same unit-cell composition as hydro­magnesite [Mg_5_(CO_3_)_4_(OH)_2_·4H_2_O] due to the pronounced long-range disorder that prevents reliable location of the additional atoms (3 C and 9 O atoms) expected in the dehydrated dypingite formula. Nonetheless, the selected and implemented crystal structure refinement methodology allowed us to determine a reliable average structure while recognizing the inherent complexity of the local structural arrangements.

The analysis of SR-PXD patterns (Fig. 8 ▸) revealed distinct behaviors of selected diffraction peaks during the dypingite hydration. The 100, 201 and 020 Bragg reflections maintained stable positions, while the 00*l* reflections showed noticeable shifts in both their position and intensity. This observation highlights the impact of hydration on various facets of dypingite crystal structure.

The unchanged 100, 201 and 020 reflections correspond to the crystallographic planes intersecting the rigid (MgO_6_)_8_–(CO_3_)_8_ and (MgO_6_)_2_–(CO_3_)_6_ networks within the lattice (Fig. 10 ▸). The interplanar distances (*d*) associated with these diffraction peaks primarily reflect the spacing between magnesium ions *within* these networks. Since the material hydration does not significantly alter the rigid internal network’s structure, the distances between Mg ions remain unchanged, resulting in the observed stability of these Bragg peaks. Notably, the interplanar distance invariance extends to the (201) planes, which cross the dypingite unit cell diagonally and intersect Mg atoms within the (MgO_6_)_2_–(CO_3_)_6_ networks (Fig. 10 ▸). While geometric considerations suggest this reflection could theoretically be influenced by changes in the (00*l*) interplanar distances, the Mg–Mg spacing within the networks exhibits negligible variation upon hydration. Consequently, the interplanar distance for (201) changes only marginally [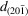 = 4.4053 and 4.4127 Å for dehydrated and hydrated dypingite, respectively], resulting in the observed stability of this reflection.

In contrast, the 00*l* peaks relate to the spacing *between* the different (MgO_6_)_8_–(CO_3_)_8_ and (MgO_6_)_2_–(CO_3_)_6_ networks. Crystallographic water molecules accumulate in the interlayer regions (Fig. 10 ▸), causing the networks to shift relative to each other. This hydration-driven interlayer expansion directly impacts the interplanar distances corresponding to the 00*l* reflections, explaining the observed changes in positions and intensities of these diffraction peaks. One can then conclude that the hydration process primarily affects the distances *between* rather than *within* the (MgO_6_)_8_–(CO_3_)_8_ and (MgO_6_)_2_–(CO_3_)_6_ networks and induces long-range disorder along the *c* axis.

**Figure 12:**
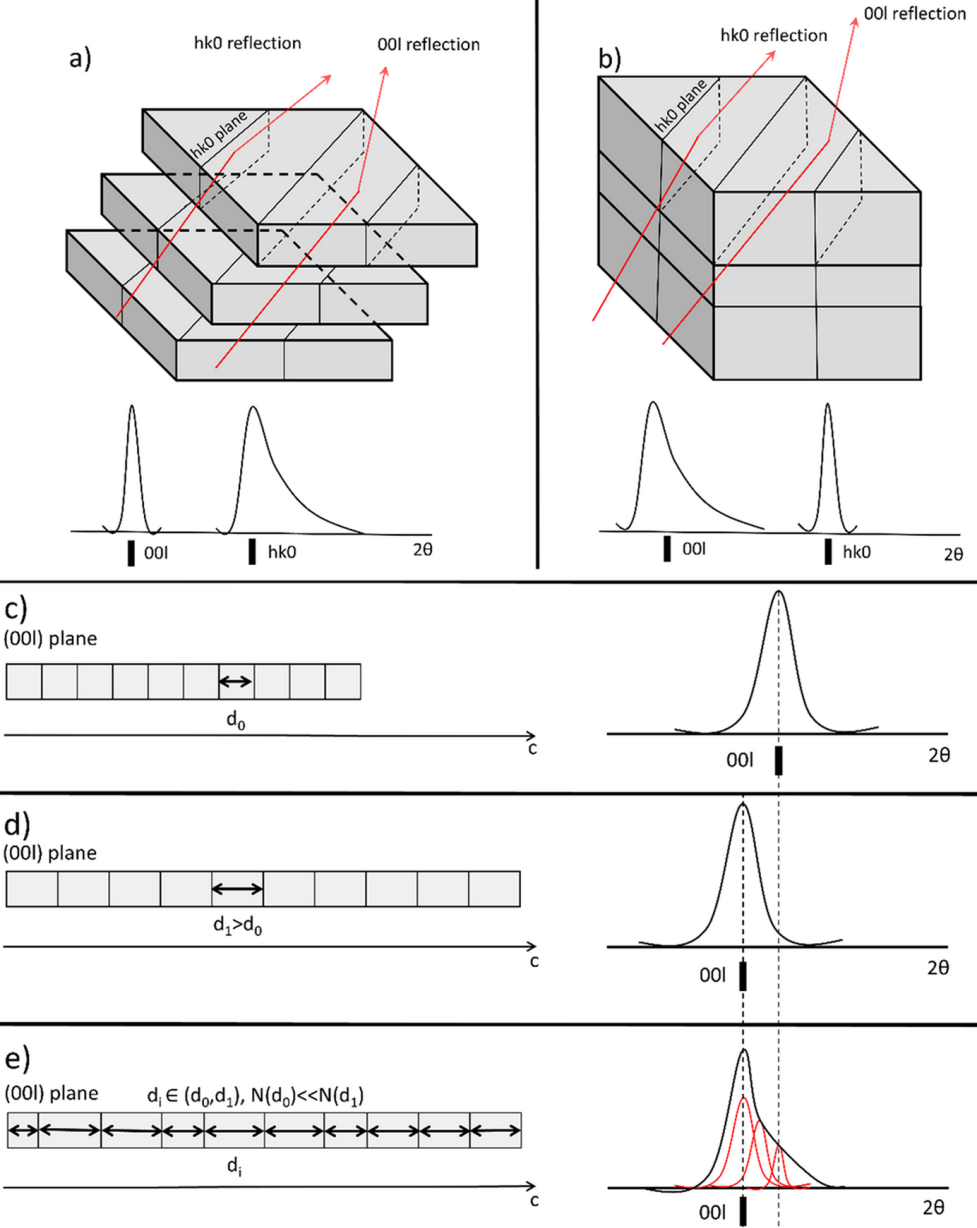
Schematic of the 00*l* Bragg peak broadening in the dypingite PXD pattern and potential underlying causes: (*a*) stacking faults with a stacking direction perpendicular to the (00*l*) lattice plane and (*b*) non-uniform uniaxial strain along the *c* axis. Influence of the uniaxial strain on the diffraction peak position and shape: (*c*) no strain, (*d*) uniform uniaxial strain and (*e*) non-uniform uniaxial strain.

This raises a crucial question: does the interlayer expansion induce Bragg peak shifts, along with their asymmetry and broadening? The increased broadness and asymmetry of the 00*l* reflections can be attributed to several factors. Potential contributors include strain gradients from uneven water molecule distribution within the crystal structure or planar defects in the form of stacking faults. While both can cause the *hkl*-dependent peak broadening, they induce it in different ways. According to Warren (1941 ▸), stacking disorder along the *c* axis leads to anisotropic broadening of the *hk*0 diffraction peaks, with sharp and unchanged 00*l* reflections [Fig. 12 ▸(*a*)]. However, the SR-PXD patterns collected in this study revealed a different behavior, with the 100, 201 and 020 Bragg peaks remaining sharp and unchanged, and the 00*l* peaks being broadened and shifted.

This unique pattern can be explained by the non-uniform expansion of the unit cell along the *c* axis caused by the increased and unevenly distributed water content in the sample volume [Figs. 12 ▸(*a*)–12 ▸(*e*)], which results in the for­mation of the irregularly distributed strain in the compound crystal structure. This behavior, when preserved and repeated over the longer range, results in the formation of the structural disorder, the presence of which is revealed by the broadened and asymmetric 00*l* Bragg peaks [Fig. 12 ▸(*e*)]. Interestingly, the process appears to be both controllable and reversible as, upon dehydration, the dypingite unit cell shrinks, while the concentration of long-range disorder decreases, leading to the sharper and less asymmetric diffraction reflections [Fig. 12 ▸(*c*)]. The humidity-responsive structural behavior of dypingite is not unique among hydrated minerals.

Notably, similar anisotropic structural changes driven by ambient humidity have been documented in the ferric sulfate tellurite hydrates, namely tamboite (*x* = 3, *y* = 2) and metatamboite (*x* = 3, *y* = 0), Fe^3+^_3_(SO_4_)(Te^4+^O_3_)_3_[Te^4+^O(OH)_2_](OH)(H_2_O)*_x_*{H_2_O}*_y_*, which undergo completely reversible transformations at room temperature (Cooper *et al.*, 2019 ▸). These minerals exhibit several striking parallels with the dypingite hydration-dependent behavior. Both systems demonstrate preferential structural changes along their stacking directions, *i.e.* the *c* axis in dypingite and the inter-slab spacing in tamboite/metatamboite. These changes occur while maintaining lateral structural coherence. In tamboite, humidity variations drive the insertion (∼50% RH) and removal (<20% RH) of interstitial (H_2_O)_4_ clusters between structural slabs, causing significant unit-cell expansion (primarily along the *a* axis, +2.484 Å) and angular changes (β angle increases by ∼10°), remarkably similar to dypingite *c* axis expansion of 2.060 Å. The anisotropic nature of these changes is reflected in the material diffraction patterns. Just as dypingite shows broadened 00*l* reflections with stable 020 and 100 Bragg peaks, the tamboite structural transformation primarily affects reflections related to inter-slab spacing, while preserving the integrity of individual structural slabs. This parallel behavior suggests a common mechanism underlying humidity-induced structural changes in layered hydrated minerals, where water molecules function as ‘structural spacers’ that modulate interlayer distances without disrupting the fundamental building units. Although the tamboite–metatamboite system demonstrates discrete structural reorganization involving the specific water cluster arrangements at defined slab positions, this differs from the continuous, non-uniform distribution of water molecules between (MgO_6_)_8_–(CO_3_)_8_ and (MgO_6_)_2_–(CO_3_)_6_ networks along the *c* axis that characterizes dypingite structural disorder.

TEM measurements carried out for the synthetic hydrated dypingite samples also revealed structural disorder propagating exclusively along the *c*-axis direction. This was demonstrated in electron diffraction patterns (EDPs) collected from a separate plate of the material in two orientations: (i) with the incident electron beam parallel to the plate surface (edge-on view) and (ii) with the beam perpendicular to the plate surface (plan view). The experimental EDPs were compared with EDPs calculated from the refined crystal structure of dehydrated dypingite.

**Figure 13:**
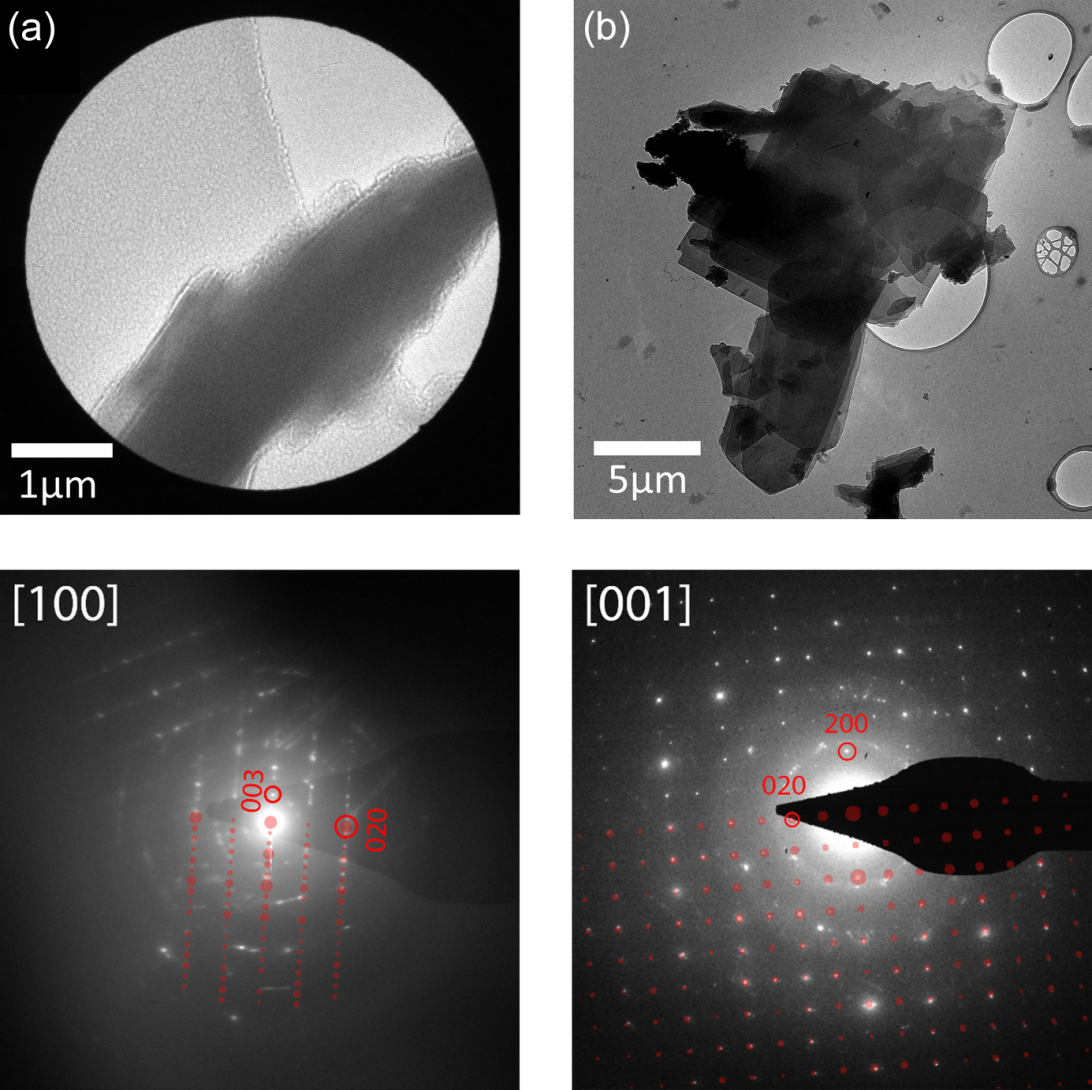
(Top) Bright-field TEM micrographs and (bottom) electron diffraction patterns of the hydrated dypingite plates measured with the electron beam (*a*) parallel and (*b*) perpendicular to the plate surface; white spots correspond to the experimental data and red spots are calculated from the refined crystal structure of dehydrated dypingite for the [100] and [001] zone axes.

In the edge-on view, the resulting EDP [Fig. 13 ▸(*a*), bottom, white spots] exhibited diffraction spots with pronounced streaking between them. The calculated EDP for the [100] zone axis [Fig. 13 ▸(*a*), bottom, red spots] showed a similar pattern of 0*kl* diffraction spots densely spaced along the [00*l*] direction, consistent with the *c* lattice constant [≃ 32.101 (5) Å]. The observed streaking between diffraction spots indicates planar defects parallel to the beam direction, arising from non-uniform *c* parameter spacing throughout the crystal. This observation provides direct evidence for structural disorder along the *c* axis, caused by the variable water content in the interlayer spaces.

In the plan view [Fig. 13 ▸(*b*)], the resulting EDP showed well defined spots arranged in a rectangular pattern, matching the calculated pattern for the [001] zone axis [Fig. 13 ▸(*b*), bottom, red spots]. The interplanar distances for the 200 and 020 diffraction spots are equal to 4.6 (3) and 4.4 (3) Å, respectively. These values are in good agreement (*i.e.* within error margins) with values determined for the 200 and 020 Bragg peaks on the basis of the SR-PXD data [*d* = 4.371 (1) and 4.184 (1) Å, respectively]. Notably, the diffuse streaking observed in the edge-on view is absent in this orientation, which is perpendicular to the direction of the planar defects. These observations confirm that the presence of structural disorder in hydrated dypingite occurs in the [00*l*] direction and are consistent with the variable hydration states affecting the interplanar distances along the *c* axis.

Another notable conclusion can be drawn from the fact that, when the dypingite plate is positioned perpendicular to the incident beam, the beam is aligned along the [001] zone axis. This indicates that the *c* axis in the dypingite plate is oriented perpendicularly to the plate surface. Preliminary observations suggest that fluctuations in ambient humidity might influence both the structural disorder within the dypingite sample and the dimensions of the material plates. However, further research is needed to conclusively validate this hypothesis. If confirmed, it could indicate that dypingite nanoplates have potential as a possible 2D material.

## 4. Conclusions

After 55 years of uncertainty, the average crystal structure of dehydrated dypingite has been determined. The main challenge with the structural characterization of this material appears to originate from a disorder within its crystal structure. The structural disorder is caused by the non-uniform increase in the spacing between the (MgO_6_)_8_–(CO_3_)_8_ and (MgO_6_)_2_–(CO_3_)_6_ networks within the dypingite crystal structure. This phenomenon is induced by material hydration and stems from the irregular distribution of water molecules within the interlayer regions in its crystal structure. This in turn results in the uneven expansion of the compound unit cells along the *c* axis observed as diffuse X-ray scattering in the collected diffraction patterns.

In this work, we have demonstrated that crystallinity of dypingite samples is affected by their origin. Synthetic dypingite was proven to have a better crystallinity than its mineral counterpart. For the synthetic dypingite samples it has also been found that the ambient humidity significantly affects the concentration of the structural disorder. By dehydrating the sample in a desiccator, a phase with a reduced amount of structural disorder was obtained, with the chemical composition Mg_5_(CO_3_)_4.5(2)_(OH)_1.02(4)_·5.0(2)H_2_O. The crystal structure of dehydrated dypingite is linked with that of hydromagnesite, and a structural model has been derived by exploiting the group–subgroup relations between these two compounds. The suggested crystal structure model of dehydrated dypingite has been successfully refined, resulting in determination of the atomic configuration and the following unit-cell parameters: *a* = 8.8424 (22) Å, *b* = 8.3920 (13) Å, *c* = 29.978 (9) Å, β = 97.781 (21)°. The refined structural model has the same unit-cell composition as hydro­magnesite [Mg_5_(CO_3_)_4_(OH)_2_·4H_2_O] since the pronounced long-range disorder prevents a reliable location of the additional atoms (3 C and 9 O atoms) expected in the dehydrated dypingite chemical formula.

The determined crystal structure of dehydrated dypingite demonstrates that the extra-water of hydration is unevenly distributed between (MgO­_6_)_8_–(CO­_3_)_8_ and (MgO­_6_)_2_–(CO­_3_)_6_ networks, altering the distances between them without affecting the atomic positions within. This causes the long-range disorder along the *c* axis and the uniaxial enlargement of the unit cells. The results obtained have shown that the uniaxial dilation of unit cells occurs in the direction perpendicular to the dypingite nanoplate surface.

This study has demonstrated that ambient humidity plays a significant role in controlling the structural properties of dypingite. By changing the humidity level around dypingite nanoparticles, one can modify the amount of structural disorder in the material volume, which in turn affects the width of the dypingite nanoplates. These properties position dypingite nanoplates alongside 2D materials like MXenes (Saleh & Hassan, 2023 ▸), offering a new perspective on the potential application of this material. We believe that the present study will spark researchers’ interest in dypingite, paving the way for broader utilization of this eco-friendly material in everyday life. Further advances in dypingite structure modeling will require additional experimental data, such as powder neutron diffraction (PND) and total scattering (PDF) measurements. These techniques would enable combined PXD–PND–PDF data refinement, allowing for accurate characterization of local atomic arrangements and coordination environments, and ultimately permitting definitive correlation of the structural formula with that determined by TGA–DSC analysis.

## Supplementary Material

Crystal structure: contains datablock(s) Dehydrated_dypingite_final. DOI: 10.1107/S1600576725007915/vb5097sup1.cif

Supporting information file. DOI: 10.1107/S1600576725007915/vb5097sup2.pdf

## Figures and Tables

**Table 1:** Reported dypingite synthesis conditions and the Bragg peak position at approximately 8° 2θ

Authors	Synthesis conditions	Synthesis reactants	8° peak position, 2θ
Montes-Hernandez *et al.* (2012 ▸)	20 °C, 24 h	Mg(OH)_2_ + CO_2_, 2 *M* NaOH as catalyst	8.85
Moore & Shabani (2016 ▸)	Stepwise synthesis: (1) 25 °C, 24 h; (2) 62 °C, 48 h; (3) 72 °C, 24 h	NaHCO_3_ + MgCl_2_·6H_2_O	8.43
Harrison *et al.* (2019 ▸)	35 °C, 57 days, final pH = 8.72	Nesquehonite (MgCO_3_·3H_2_O) in NaCl and NaHCO_3_ solution	8.44
Tanaka *et al.* (2019 ▸)	15 °C, 21 days, final pH = 9.5	Na_2_CO_3_·10H_2_O + MgCl_2_·6H_2_O	8.38
Yamamoto *et al.* (2022 ▸)	60 °C, 2 h, final pH = 11	Na_2_CO_3_·10H_2_O + MgCl_2_·6H_2_O	8.26
Ballirano *et al.* (2013 ▸)	After 24 h precipitate was filtered, residual solution was settled at 27 °C for 230 days	MgCl_2_·6H_2_O + CO_2_, 25% NH_3_(aq) for regulation of pH in the range 8–9	8.12
Raade (1970 ▸)	First reported mineral dypingite; collected from the serpentine–magnesite deposit at Dypingdal, Snarum, South Norway	–	8.33

**Table 2:** Unit-cell parameters of mineral dypingite, as obtained in the present study, along with the earlier reported values for dypingite, hydro­magnesite and nesquehonite

	SG	*a* (Å)	*b* (Å)	*c* (Å)	β (°)	*V* (Å^3^)	χ^2^
Dypingite†	*P*2_1_	8.8593 (2)	8.3846 (5)	32.655 (4)	97.801 (8)	2402.7 (5)	6.95 (1)
	*P*2	8.8532 (4)	8.3831 (5)	32.587 (3)	97.648 (5)	2397.2 (6)	6.87 (1)
Dypingite, Mg_5_(CO_3_)_4_(OH)_2_·5H_2_O‡	*P*2/*m*	10.336 (1)	33.793 (2)	35.706 (2)	114.55 (5)	11345 (1)	1.56 (7)
Hydro­magnesite, Mg_5_(CO_3_)_4_(OH)_2_·4H_2_O§	*P*2_1_/*c*	10.11 (1)	8.97 (7)	8.38 (3)	114.6 (4)	690.9 (9)	–
Nesquehonite, MgCO_3_·3H_2_O¶	*P*2_1_/*m*	7.701 (1)	5.365 (2)	12.126 (1)	90.41 (2)	500.983 (3)	–

† Mineral, this study.

‡ Synthetic (Ballirano *et al.*, 2013 ▸).

§ Mineral (Akao *et al.*, 1974 ▸).

¶ Mineral (Giester *et al.*, 2000 ▸).

**Table 3:** Comparison of unit-cell parameters: hydrated and dehydrated synthetic dypingite; *ISODISTORT*-calculated model of dehydrated dypingite; and literature values for hydro­magnesite

	SG	*a* (Å)	*b* (Å)	*c* (Å)	β (°)	*V* (Å^3^)
Hydrated†	*P*2­_1_	8.8403(7)	8.3766(3)	32.101(5)	97.100(9)	2358.9(4)
Dehydrated†	*P*2_1_	8.8317(2)	8.3734(1)	30.041(9)	98.040(2)	2198.4(8)
Calculated‡	*P*2_1_	8.3886(1)	8.9536(1)	27.967(5)	98.516(5)	20774(2)
Hydro­magnesite§	*P*2_1_/*c*	10.11	8.97	8.38	114.6	690.8

† Synthetic dypingite.

‡ Dypingite model generated by *ISODISTORT*.

§ Mineral (Akao *et al.*, 1974 ▸).

## Data Availability

The supplementary crystallographic data for this paper are deposited on https://dataverse.no/ and can be accessed via the following link: https://doi.org/10.18710/PABU7Q. The crystallographic information file of dehydrated dypingite is deposited with the Cambridge Crystallographic Data Centre under the deposition number CSD 2422228 and can be accessed free of charge via https://www.ccdc.cam.ac.uk/data_request/cif. Sup­porting information is also available from IUCr Journals (https://journals.iucr.org/), the Wiley Online Library or the author.
