# The crystal structure of dypingite: understanding the long-range disorder. Corrigendum

**DOI:** 10.1107/S1600576726002438

**Published:** 2026-03-11

**Authors:** Anton Sednev-Lugovets, Yang Lu, Ørnulv Vistad, Patricia Almeida Carvalho, Alexander Missyul, Håkon Austrheim, Henrik Friis, Matylda N. Guzik

**Affiliations:** a Department of Technology Systems University of Oslo Gunnar Randers vei 19 Kjeller2007 Norway; b Natural History Museum University of Oslo Blindern Oslo1172 Norway; c SINTEF Industry, Forskningsveien 1, Oslo0374, Norway; d CELLS-ALBA Synchrotron, Carrer de la Llum 2-26, Barcelona08290, Spain; e Department of Geosciences University of Oslo Sem Saelands vei 1 Oslo0371 Norway; Australian Synchrotron, ANSTO, Australia

**Keywords:** dypingite, structural disorder, uniaxial elongation, magnesium hy­droxy carbonates, long-range disorder

## Abstract

An error in the article by Sednev-Lugovets *et al.* [*J. Appl. Cryst.* (2025), **58**, 1908–1919] is corrected.

The article by Sednev-Lugovets *et al.* (2025 ▸) contains a factual error in the description of the TGA–DSC results: in the discussion of Fig. 6, the thermal effect at approximately 62 °C is described as exothermic, whereas it is in fact endothermic. The second paragraph of Section 3.2 should read as follows:

The compositional difference between the hydrated and dehydrated samples of synthetic dypingite was studied by thermogravimetric analysis, coupled with differential scanning calorimetry (TGA–DSC). On heating, the two samples underwent similar decomposition stages (Fig. 6) with peak temperature (*T*_p_) events at 120 and 245 °C, corresponding to the release of water from the material. The third process at *T*_p_ = 433 °C was associated with the sample decarbonation. While these findings are consistent with previous reports, the TGA–DSC curve of the hydrated sample demonstrated an additional decomposition step, with a pronounced *endothermic* effect at *T*_p_ = 62 °C (Raade, 1970; Suzuki & Ito, 1973; Canterford *et al.*, 1984; Yamamoto *et al.*, 2022; Frost *et al.*, 2008). This was associated with desorption of water previously accommodated by the sample during the hydration process.
