# Supplementary material for: The crystal structure of dypingite: understanding the long-range disorder
Source: J Appl Crystallogr. 2025 Oct 10;58(Pt 6):1908–19. doi: 10.1107/S1600576725007915 (PMC12810463; doi:10.1107/S1600576725007915)
Supplement: Supplementary file 2 [file j-58-01908-sup2.pdf]

JOURNAL OF
APPLIED
CRYSTALLOGRAPHY

Volume 58 (2025)

Supporting information for article:

The crystal structure of dypingite: Understanding the long-range disorder

Anton Sednev-Lugovets, Yang Lu, Ørnulv Vistad, Patricia Almeida Carvalho, Alexander Missyul, Håkon Austrheim, Henrik Friis and Matylda Natalya Guzik

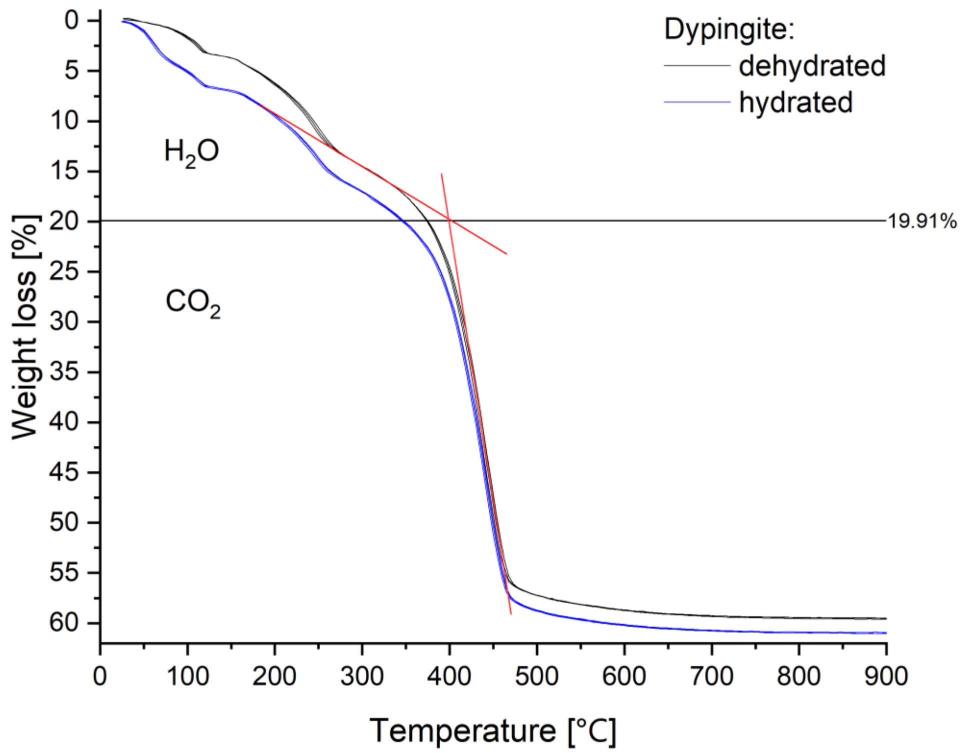

Figure S1 Determination of the chemical composition of hydrated and dehydrated dypingite based on the TGA-DSC data. Black lines – dehydrated dypingite, blue lines – hydrated dypingite, red lines are tangential lines.

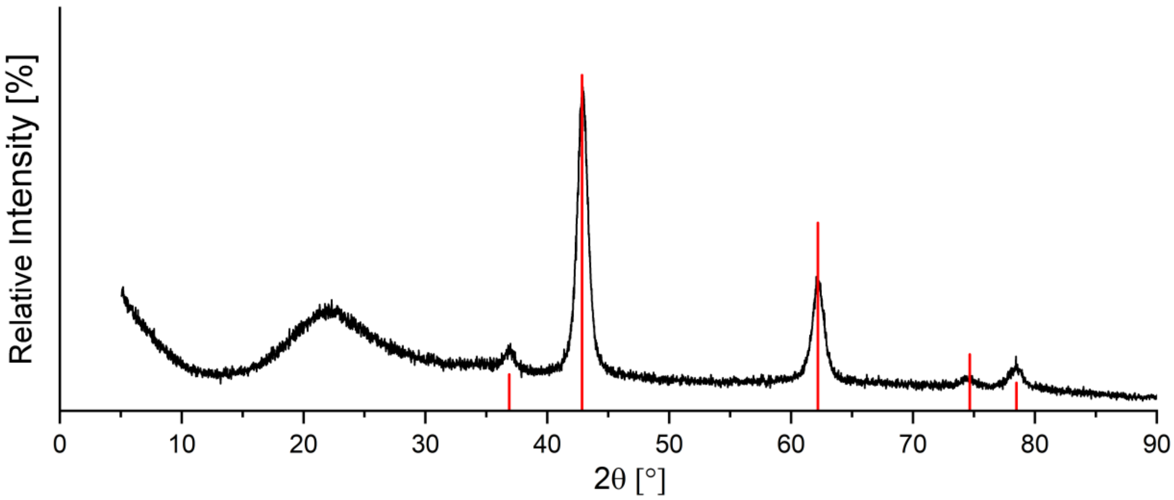

Figure S2 PXD pattern of the dypingite sample after the TGA-DSC measurement. Black line – the pattern of the sample's residue, red vertical lines – reference positions of the MgO diffraction peaks.

Table S1 The estimated weight percentage and calculated chemical formulas of hydrated and dehydrated dypingite.

Samples#	weight percentage CO2, wt.%	weight percentage H2O, wt.%	weight percentage MgO, wt.%	weight loss, %
Dehydrated#1	39.57	19.94	40.49	59.51
Dehydrated#2	39.36	20.26	40.38	59.62
Dehydrated#3	40.01	19.53	40.46	59.54
Average dehydrated	39.6(15)	19.9(7)	40.4(15)	59.56(5)
Hydrated#1	38.55	22.49	38.96	61.04
Hydrated#2	38.62	22.41	38.97	61.03
Hydrated#3	38.36	22.58	39.06	60.94
Average hydrated	38.5(14)	22.5(8)	39.0(14)	61.00(5)

Table S2 Reported molar percentage and measured weight losses reported compared with ideal dypingite and hydromagnesite formulae.

	CO_2, mol%	H_2O, mol%	MgO, mol%	weight loss, %
(Raade, 1970)	27.18	41.48	31.34	58.80
(J. Suzuki & M. Ito, 1973)	21.03	48.49	30.47	59.43
(Canterford & Tsambourakis, 1984)	24.69	42.88	32.43	58.32
(Yamamoto et al., 2022)	26.87	39.93	33.20	58.70
(Lu *et al.*, 2025) Sample (05HR-1)	29.53	32.08	38.39	54.82
(Lu *et al.*, 2025) Sample (07-2)	26.20	43.24	30.55	61.07
Hydrated dypingite, present study	28.3(3)	40.4(4)	31.3(1)	61.00(5)

Dehydrated dypingite, present study	29.9(1)	36.7(1)	33.34(2)	59.60(5)
"Ideal" dypingite $Mg_5(CO_3)_4(OH)_2{\cdot}5H_2O$	26.66	40.0	33.33	58.5
"Ideal" hydromagnesite $Mg_5(CO_3)_4(OH)_2{\cdot}4H_2O$	28.57	35.71	35.71	56.90

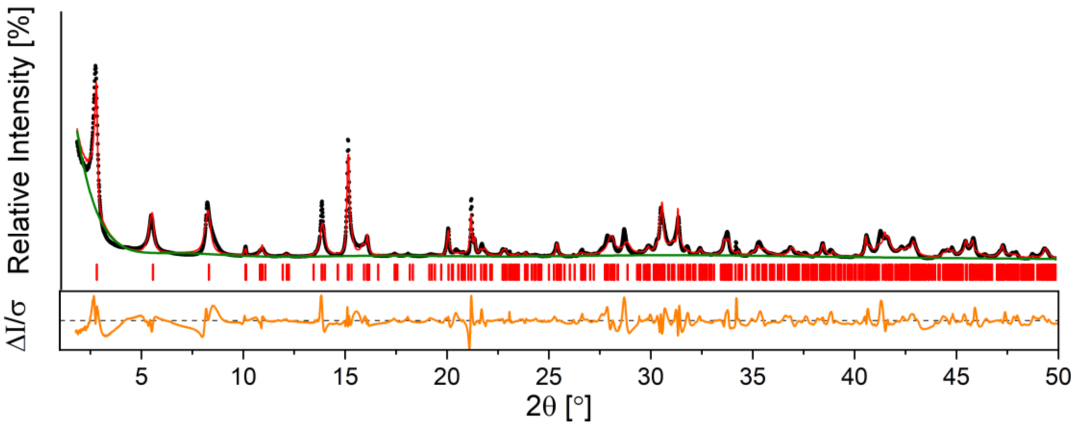

Figure S3 Le Bail refinement results of SR-PXD data for mineral dypingite (SG = *P*2). Black dots, red lines, and vertical red bars represent the experimental and calculated powder diffraction profiles, and calculated positions of the Bragg peaks, correspondingly. The orange line illustrates the difference between experimental and calculated SR-PXD data; λ = Cu Kα_1.

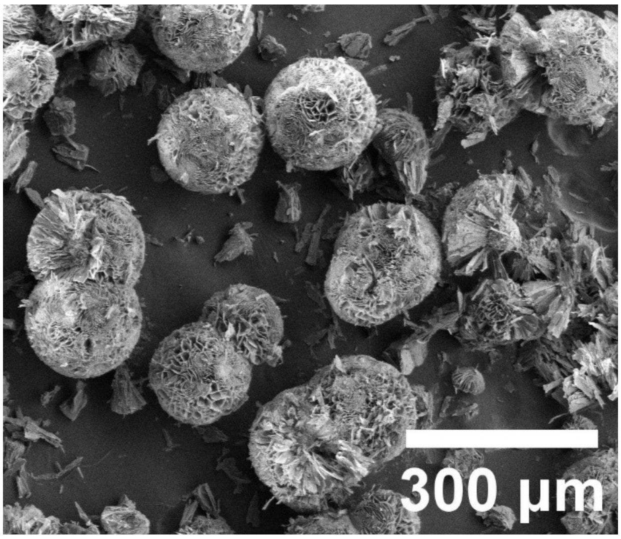

Figure S4 SEM image of synthetic dypingite nanoflowers synthesized at 40°C for 28 days at atmospheric pressure, subsequently kept at 80% RH for 10 days.

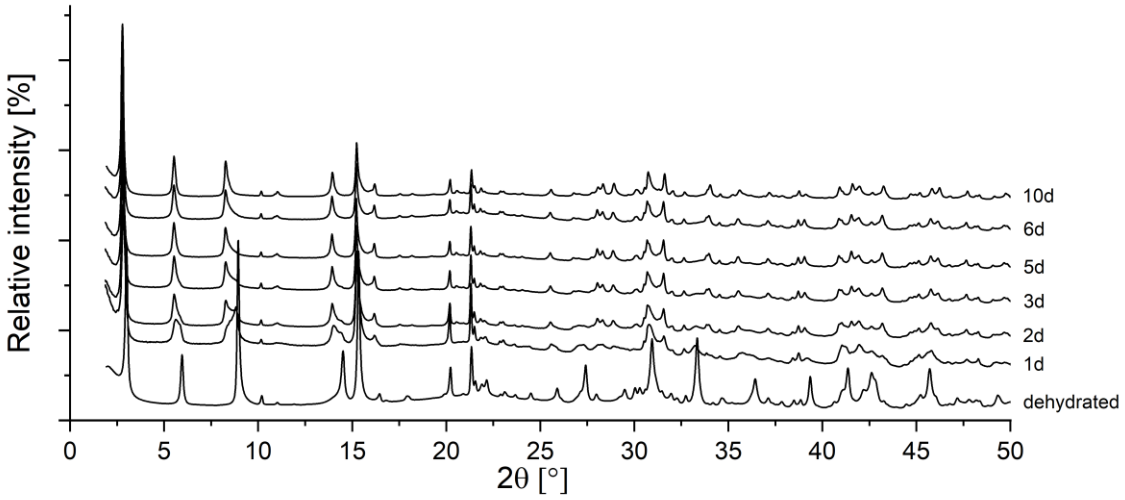

Figure S5 SR-PXD data collected upon hydration of the fully dehydrated dypingite in a high humidity atmosphere (80% RH, 22°C). The numbers at the right-hand side indicate number of hydration days.

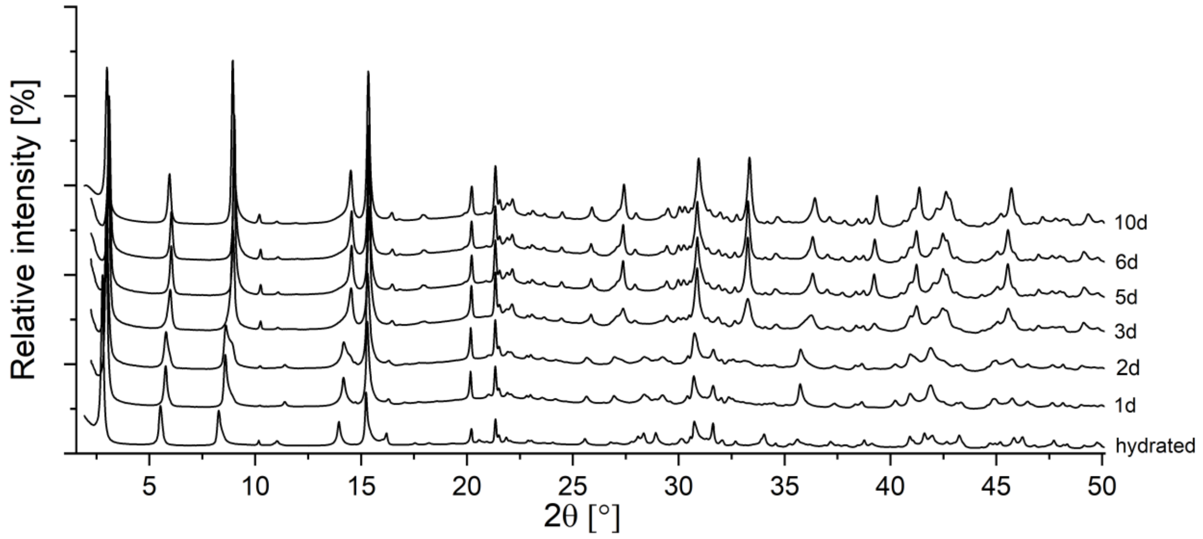

Figure S6 SR-PXD data collected upon reverse transition of the sample presented in S5. The dehydration took place in a low-humidity atmosphere (20% RH, 22°C). The numbers at the right-hand side indicate number of days the sample was kept in a desiccator.

Table S3 Atomic coordinates for the model (ISODISTORT generated) and refined crystal structure of dehydrated dypingite.

	Crystal structure model			Refined crystal structure		
atom name	x	y	z	x	y	z
Mg1	0.003900	0.366100	0.385000	-0.061(14)	0.392(12)	0.376(4)
Mg2	0.337230	0.366100	0.051670	0.382(14)	0.311(13)	0.036(4)
Mg3	0.329430	0.866100	0.281670	0.499(11)	0.090(12)	0.378(4)
Mg4	0.503900	0.006100	0.385000	0.346(11)	0.856(14)	0.277(4)
Mg5	0.170570	0.006100	0.718330	0.130(12)	-0.006(14)	0.715(4)
Mg6	0.837230	0.006100	0.051670	0.888(13)	0.015(10)	0.065(4)
Mg7	0.128730	0.500000	0.615270	0.123(12)	0.484(14)	0.631(4)
Mg8	0.204600	0.000000	0.051400	0.215(14)	-0.167(12)	0.075(4)
Mg9	0.462070	0.500000	0.281930	0.410(14)	0.444(13)	0.253(4)
Mg10	0.371270	0.372200	0.384730	0.353(13)	0.324(14)	0.372(4)
Mg11	0.037930	0.372200	0.718070	0.001(15)	0.434(12)	0.722(4)
Mg12	0.704600	0.372200	0.051400	0.787(14)	0.378(13)	0.039(4)
Mg13	0.250000	0.436100	0.500000	0.273(13)	0.486(14)	0.500(4)
Mg14	0.583330	0.436100	0.166670	0.591(13)	0.390(11)	0.144(4)
Mg15	0.083330	0.936100	0.166670	0.095(13)	0.973(14)	0.178(4)
C1	0.004560	0.160800	0.469540	-0.03(3)	0.202(28)	0.469(7)
C2	0.337900	0.160800	0.136200	0.32(4)	0.198(23)	0.137(7)
C3	0.328770	0.660800	0.197130	0.300(25)	0.67(4)	0.167(7)
C4	0.504560	0.211400	0.469540	0.55(4)	0.260(25)	0.499(7)
C5	0.171230	0.211400	0.802870	0.19(3)	0.185(24)	0.802(6)
C6	0.837900	0.211400	0.136200	0.85(3)	0.20(4)	0.155(6)
C7	0.160030	0.102500	0.342970	0.215(20)	0.103(24)	0.339(12)
C8	0.493370	0.102500	0.009630	0.512(23)	0.018(19)	0.030(12)
C9	0.173300	0.602500	0.323700	0.218(19)	0.623(25)	0.345(6)
C10	0.660030	0.269700	0.342970	0.670(21)	0.275(23)	0.303(8)
C11	0.326700	0.269700	0.676300	0.391(20)	0.263(17)	0.686(13)
C12	0.993370	0.269700	0.009630	1.060(20)	0.184(23)	-0.003(12
O1	0.217470	0.453300	0.426030	0.174(14)	0.422(21)	0.429(5)
O2	0.550800	0.453300	0.092700	0.571(17)	0.485(17)	0.072(5)

O3	0.115870	0.953300	0.240630	0.195(15)	1.030(17)	0.252(4)
O4	0.282530	0.418900	0.573970	0.310(16)	0.463(21)	0.582(4)
O5	0.615870	0.418900	0.240630	0.639(17)	0.463(24)	0.223(4)
O6	0.050800	0.918900	0.092700	0.006(18)	0.868(17)	0.106(4)
O7	0.032500	0.830200	0.417500	0.122(19)	0.887(23)	0.418(7)
O8	0.365830	0.830200	0.084170	0.437(16)	0.845(23)	0.098(9)
O9	0.300830	0.330200	0.249170	0.135(16)	0.405(22)	0.241(8)
O10	0.467500	0.042000	0.582500	0.506(22)	0.037(17)	0.609(9)
O11	0.800830	0.042000	0.249170	0.766(22)	0.047(25)	0.236(9)
O12	0.134170	0.042000	0.915830	-0.014(23)	-0.007(21)	0.912(8)
O13	0.133530	0.008700	0.581370	0.127(24)	0.118(20)	0.598(8)
O14	0.199800	0.508700	0.085300	0.198(19)	0.473(21)	0.062(7)
O15	0.466870	0.008700	0.248030	0.422(26)	0.070(19)	0.230(7)
O16	0.633530	0.363500	0.581370	0.615(23)	0.341(18)	0.603(8)
O17	0.966870	0.363500	0.248030	0.891(27)	0.405(20)	0.215(5)
O18	0.300200	0.363500	0.914700	0.277(20)	0.348(21)	0.903(6)
O19	0.101900	0.254000	0.497900	0.079(18)	0.302(19)	0.503(7)
O20	0.435230	0.254000	0.164570	0.345(17)	0.342(25)	0.127(8)
O21	0.231430	0.754000	0.168770	0.135(26)	0.728(18)	0.155(7)
O22	0.601900	0.118200	0.497900	0.613(24)	0.156(18)	0.544(5)
O23	0.268570	0.118200	0.831230	0.203(18)	0.063(20)	0.828(7)
O24	0.935230	0.118200	0.164570	0.868(21)	0.090(27)	0.196(7)
O25	0.046100	0.559300	0.504200	0.026(17)	0.580(24)	0.498(9)
O26	0.287230	0.059300	0.162470	0.202(23)	0.238(19)	0.172(8)
O27	0.379430	0.559300	0.170870	0.375(21)	0.577(27)	0.131(8)
O28	0.453900	0.312900	0.495800	0.551(15)	0.449(24)	0.510(8)
O29	0.120570	0.312900	0.829130	0.105(19)	0.321(27)	0.811(8)
O30	0.787230	0.312900	0.162470	0.805(21)	0.282(23)	0.191(8)
O31	0.050200	0.683600	0.574000	0.149(21)	0.715(15)	0.582(5)
O32	0.283130	0.183600	0.092670	0.319(23)	0.095(18)	0.092(5)
O33	0.383530	0.683600	0.240670	0.389(25)	0.710(15)	0.223(5)
O34	0.449800	0.188600	0.426000	0.447(22)	0.228(20)	0.444(4)
O35	0.116470	0.188600	0.759330	0.153(23)	0.229(17)	0.756(5)

O36	0.783130	0.188600	0.092670	0.901(22)	0.262(14)	0.103(4)
O37	0.172670	0.243000	0.356730	0.126(13)	0.266(19)	0.339(7)
O38	0.506000	0.243000	0.023400	0.578(14)	0.197(16)	0.025(7)
O39	0.160670	0.743000	0.309930	0.165(16)	0.731(18)	0.303(7)
O40	0.672670	0.129200	0.356730	0.644(16)	0.112(24)	0.324(4)
O41	0.339330	0.129200	0.690070	0.369(14)	0.101(18)	0.699(7)
O42	0.006000	0.129200	0.023400	0.083(16)	0.002(17)	0.020(5)
O43	0.035100	0.039600	0.336400	0.043(18)	0.098(20)	0.325(5)
O44	0.368430	0.039600	0.003070	0.406(15)	-0.145(22)	0.027(5)
O45	0.298230	0.539600	0.330270	0.289(21)	0.505(20)	0.316(5)
O46	0.535100	0.332600	0.336400	0.518(18)	0.302(22)	0.310(5)
O47	0.201770	0.332600	0.669730	0.238(20)	0.334(21)	0.692(5)
O48	0.868430	0.332600	0.003070	0.876(19)	0.195(18)	-0.013(5)
O49	0.304930	0.035700	0.333470	0.333(17)	-0.038(17)	0.341(6)
O50	0.638270	0.035700	0.000130	0.649(18)	-0.039(22)	0.021(7)
O51	0.028400	0.535700	0.333200	0.056(18)	0.577(21)	0.357(5)
O52	0.804930	0.336500	0.333470	0.732(14)	0.448(24)	0.334(5)
O53	0.471600	0.336500	0.666800	0.463(14)	0.429(18)	0.684(5)
O54	0.138270	0.336500	0.000130	0.166(19)	0.310(17)	-0.014(6)
H1	0.210670	0.540100	0.423330			
H2	0.544000	0.540100	0.090000			
H3	0.122670	0.040100	0.243330			
H4	0.289330	0.332100	0.576670			
H5	0.622670	0.332100	0.243330			
H6	0.044000	0.832100	0.090000			
H7	0.026330	0.803100	0.445670			
H8	0.359670	0.803100	0.112330			
H9	0.307000	0.303100	0.221000			
H10	0.473670	0.069100	0.554330			
H11	0.807000	0.069100	0.221000			
H12	0.140330	0.069100	0.887670			
H13	0.009670	0.252100	0.596330			
H14	0.323670	0.752100	0.070330			

H15	0.343000	0.252100	0.263000			
H16	0.509670	0.120100	0.596330			
H17	0.843000	0.120100	0.263000			
H18	0.176330	0.120100	0.929670			
H19	0.086670	0.025100	0.548330			
H20	0.246670	0.525100	0.118330			
H21	0.420000	0.025100	0.215000			
H22	0.586670	0.347100	0.548330			
H23	0.920000	0.347100	0.215000			
H24	0.253330	0.347100	0.881670			
H25	0.235000	0.021100	0.581000			
H26	0.098330	0.521100	0.085670			
H27	0.568330	0.021100	0.247670			
H28	0.265000	0.851100	0.419000			
H29	0.068330	0.351100	0.247670			
H30	0.598330	0.851100	0.085670			

Table S4 Lattice constants of dehydrated dypingite as obtained for the model and refined crystal structures.

Dypingite unit cell parameters	Space Group	a, Å	b, Å	c, Å	β, °	V, Å^3
Model	P21	8.8311(2)	8.3734(1)	30.041(9)	98.040(2)	2198.4(8)
Refined	P21	8.8424(22)	8.3920(13)	29.978(9)	97.781(21)	2204.1(8)
